# Clinical challenges, controversies, and regional strategies in snakebite care in India

**DOI:** 10.1016/j.lansea.2025.100598

**Published:** 2025-05-15

**Authors:** Siju V. Abraham, Deo Mathew, Aravind Sreekumar, Akhil V. George, Vijay Chanchal, Purushothaman Kuzhikkathu Kandiyil, Punchalil Chathappan Rajeev, Udaya Bhaskaran Valuvil, Jayesh Kumar, Kuruvath Bahuleyan Mohan, Joe Thomas, Manu Ayyan, Sandeep Das, Freston Marc Sirur, Indira Madhavan, Aboobacker Mohamed Rafi, Pradeoth Korambayil Mukundan, Kartik Sunagar, Vimal Krishnan S, Babu Urumese Palatty

**Affiliations:** a Department of Emergency Medicine, Jubilee Mission Medical College and Research Institute, Thrissur, Kerala, India; b Department of Pediatrics, MES Academy of Medical Sciences, Perinthalmanna, Kerala, India; c Department of General Medicine, Malabar Medical College & Research Centre, Kozhikode, Kerala, India; d Department of General Medicine, Government Medical College, Kozhikode, Kerala, India; e Department of General Medicine, Jubilee Mission Medical College & Research Institute, Thrissur, Kerala, India; f Department of Emergency Medicine, Jawaharlal Institute of Postgraduate Medical Education & Research, Puducherry, India; g Department of Zoology, University of Calicut, Thenhipalam, Kerala, India; h Kasturba Medical College, Manipal, Manipal Academy of Higher Education (MAHE), Manipal, Karnataka, India; i Department of General Medicine, Government Medical College, Ernakulam, Kerala, India; j Department of Transfusion Medicine, Jubilee Mission Medical College and Research Institute, Thrissur, Kerala, India; k Department of Plastic Surgery, Burns and Hyperbaric Medicine, Jubilee Mission Medical College & Research Institute, Thrissur, Kerala, India; l Evolutionary Venomics Lab, Indian Institute of Science, Bengaluru, Karnataka, India

**Keywords:** Snakebite, Antivenins, Venoms, Capillary leak syndrome, Regional health, One health

## Abstract

Snakebite envenomation remains a significant public health issue, particularly in southeast Asia, where diverse venomous snake species and resource-limited healthcare settings complicate effective management. This Health Policy employed thematic analysis of a panel discussion involving multidisciplinary experts with over 300 years of combined experience. Four key themes were identified: (i) the evolution of snakebite treatment paradigms, (ii) clinical and procedural challenges, (iii) debates over controversial practices, and (iv) the role of policy and research in improving outcomes. The findings emphasise the need for region-specific antivenoms, enhanced peripheral healthcare capabilities, and evidence-based treatment protocols. This work provides actionable insights to inform health policy, guide targeted training initiatives, and prioritise research on neglected areas in the management of snakebite.

## Introduction

Snakebite envenomation remains a significant public health challenge in India, particularly in rural areas where access to timely and appropriate medical care is limited.^1^ The complexities of snakebite management are compounded by the diverse species of venomous snakes present in the region, each with varying effects on the human body. Despite advancements in antivenom production and snakebite care protocols, there remains considerable debate and variability in treatment approaches, particularly for species like Hump-nosed pit viper (*Hypnale hypnale*) that are not covered by the Indian polyvalent antivenom (IPAV).^2^

The Snake Bite Life Support (SBLS) workshop, held as part of ongoing efforts to enhance snakebite management within a One Health framework, to improve snakebite management, brought together a panel of experts with over 300 years of collective experience in treating snakebite cases.^3^ This panel discussion, titled “Core Principles and Controversies in Snake Bite Management”, aimed to explore the evolving treatment paradigms, the challenges faced by clinicians, and the need for region-specific antivenom strategies. The discussion also focused on the current consensus guidelines and the controversies that still exist in the field, providing valuable insights into the complexities of managing snakebite envenomation in the region.

## Methods

### Context and setting

The study was conducted as part of a national workshop on snakebite management, held at [a tertiary care institute] May 17–19, 2024. The workshop featured a 90-min panel discussion moderated by the principal investigator (SVA), with predefined questions addressing audience-submitted queries through Whatsapp groups. The discussion took place in a structured environment, with experts from various medical and scientific disciplines sharing their insights on snakebite management practices and challenges. This structured format was intentionally ensured so that the discussion remained focused on practical challenges, acknowledging that the structured nature might hinder spontaneous emergence of themes or inter-participant dynamics.

### Design

This study employed a qualitative research design within an interpretivist paradigm, utilising thematic analysis to explore the perspectives and experiences of experts in snakebite management. The thematic analysis allowed for the identification and interpretation of patterns and themes within the data, providing in-depth insights into the complexities of snakebite treatment and management. Thematic analysis was chosen for its flexibility and ability to systematically identify and interpret patterns within diverse qualitative data. While panel discussions differ from focus group discussions or in-depth interviews, the use of a verbatim transcript enabled analysis, aligning with established methodologies. Ethical approval for the study was obtained from an appropriate ethics review board (IEC: 108/24/IEC/JMMC&RI). All participants provided informed consent for their contributions to be recorded and used for research purposes.

### Sampling strategy

A purposive sampling strategy was employed to select the panellists, with extensive experience and expertise in snakebite management. The selection process was guided by the following criteria by the organising team:

Candidates with notable published research, clinical expertise, or active roles in policymaking related to snakebite management were prioritised. Panellists were selected based on their years of practice and expertise in snakebite management and related fields. The field of expertise of the panellists was evaluated to ensure alignment with the study's objectives, guaranteeing a comprehensive and balanced representation of diverse perspectives.

The process commenced with the identification of pioneers in the field and individuals recognised for their significant contributions to snakebite care aligning with the above criteria. Team discussions were conducted to refine the selection further, weighing the professional achievements and relevance of potential panellists. Additionally, panellists were chosen for their reputation as key figures in the domain, ensuring an engaging and insightful session for participants while fostering diverse perspectives essential to the study's objectives.

### Researcher characteristics and reflexivity

The research team consisted of experts with diverse backgrounds in snakebite management. The panellists, who are also the authors, are recognised leaders in relevant fields, including general physicians (KBM, UBV, JT), a paediatrician (PKK), emergency medicine physicians (RPC, MA, FMS), a wilderness medicine expert (FMS), national policymakers (FMS, MA), a toxicologist (IM), a haematologist (AMR), and a zoologist (SD). Each has at least 15 years of experience in treating snakebites, bringing over 300 years of combined clinical experience to the discussion (see Supplementary Information S1: Panellist profile).

Although familiar with each other's work, the panellists had not previously collaborated, requiring a conscious effort to ensure open, unbiased dialogue. Reflexive practices were employed to prevent prior knowledge or assumptions from influencing the analysis, ensuring that diverse perspectives were adequately captured and represented in the study. Coding and theme development were reviewed independently by multiple researchers, ensuring that diverse perspectives were adequately captured and reducing the potential for bias.

### Data management and analysis

Data collection involved video recordings of the 90-min panel discussion using a Canon EOS 60D camera, with a Canon EOS 1500D as backup. Audio and video were stored as MP4 files and securely backed up. Field notes were taken during the session using Microsoft OneNote on a Surface Pro. Audio recordings were transcribed verbatim by AG, verified by DM, and cross-checked for accuracy by VC. The transcripts were shared with panellists for revision to ensure accuracy. Initial coding of the transcripts was done using Microsoft Excel, with additional codes generated using OpenAI's GPT-4 and manually assigned by AG and SVA, then verified by AS. Supporting quotes were organised under relevant themes and subthemes, with overlapping statements assigned multiple codes. In instances where a sentence or segment was relevant to multiple themes, we duplicated the quote under each applicable code to ensure comprehensive representation of the data (Supplementary Information S2: Coded quotes). Frequency summaries were used descriptively to illustrate recurring themes, but thematic prioritisation was based on the depth and relevance of insights, rather than frequency alone. Thematic analysis followed Braun and Clarke's six-phase framework.^4^ Data were securely stored in password-protected cloud storage (Google Drive).

## Results

### Thematic analysis of the panel discussion on snakebite management

The thematic analysis identified four key themes. The first theme focused on the evolution of snakebite treatment, transitioning from unspecialised to specialised care, setting the foundation for subsequent discussions. This shift highlighted the importance of specialised care in addressing snakebite management, leading to the second theme: the clinical and procedural challenges that emerge in treating snakebite cases. From these challenges, the third theme surfaced, covering the debates, controversies, and doubts among the expert panellists. Finally, the fourth theme addressed the role of policy and research in shaping future approaches to snakebite management.

### Theme 1: evolution of snakebite treatment paradigms (ES)

This theme captures the progressive changes in snakebite management, focussing on antivenom use, the establishment of specialised care units, and shifts in diagnostic practices.

• **ES-AV (Historical changes in antivenom use)**: The approach to antivenom has evolved from limited and low-dose use in the 1970s to a standardised 10-vial treatment across snake species. “*The serious nature of pit viper bites was fully acknowledged in 2006, marking a major shift in management*” (KBM).^5^ Recognising the ineffectiveness of antivenom for pit viper bites helped reduce wastage and unnecessary administration.
• **ES-SC (Advancements in specialised care)**: Specialised snakebite care units were established in response to high mortality rates from complications such as capillary leak syndrome and renal failure. The introduction of haemodialysis and ventilatory support in the 1970s was a turning point, significantly reducing mortality (“*The introduction of dialysis and ventilatory support greatly reduced mortality rates …* ”–KBM).
• **ES-DX (Historical changes in diagnosis)**: Early diagnostic challenges, such as misidentifying species, compounded treatment issues (UB, KBM). The limited number of experts and lack of literature made it difficult to distinguish between species and species specific syndromes during that period (“*Initially thought to be a subspecies of the saw-scaled viper … later identified as a Hump-Nosed Pit Viper*”–KBM).

### Theme 2: clinical and procedural challenges in snakebite management (CP)

This theme addresses the various clinical and procedural difficulties encountered in managing snakebites, including challenges specific to high-volume centres.

#### CP-C (challenges in snakebite management)

Challenges in snakebite management span the entire continuum of care, from prehospital treatment to hospital care. In rural areas, “patients often visit traditional healers”, leading to delays in presentation (RPC). Once at the hospital, variations in case presentation and inconsistent management complicate treatment, particularly when referral systems fail.^6^^,^^7^ Peripheral centres frequently lack resuscitation support and clarity on antivenom dosage, leading to confusion and sometimes non-administration of anti-snake venom (ASV). Early ‘referral to better-equipped centres’ is often seen as the ‘safest option’ (Audience). Additionally, managing allergic reactions, coagulopathy, capillary leak syndrome, and other complications remains a significant challenge in these settings.

Allergy and anaphylaxis, issues in managing venom-induced consumptive coagulopathy (VICC), capillary leak syndrome (CLS), thrombotic microangiopathy, renal failure, and special situations like stroke and paediatric snakebite management, were the other topics discussed.

#### CP-AX (allergy and anaphylaxis)

Non administration of antivenom especially in the peripheral centres “due to concerns about anaphylaxis” was discussed (RPC). “Anaphylaxis can occur with any amount of antivenom”, so it was suggested that peripheral centres, when indicated, should consider administering the full required dose, with the patient monitored before transfer (FMS).

#### CP-VIC (venom-induced coagulopathy)

“*Venom-induced consumption coagulopathy can lead to systemic manifestations such as stroke, myocardial infarction, and acute kidney injury*” (FMS).^8^ In certain regions *H. hypnale* bites are associated with “*more severe complications and higher mortality rates*” due to coagulopathy (FMS).^9^ The complexity of coagulopathy in snakebites is heightened by the fact that “*snake venom contains both procoagulants and anticoagulants, and the dominant factors determine the presentation of symptoms*” (UB).^8^ This raises critical questions in treatment, such as whether “*anticoagulation is an appropriate treatment option for hemispheric stroke*” (UB) or if blood component therapy, while considered the next best option after antivenom, can “*lead to more complications than benefits if used without a clear indication*” (JT).

#### CP-TMA (thrombotic microangiopathy)

“*Thrombotic microangiopathy (TMA) is not uncommon in snakebite cases in India, often presenting with atypical symptoms*” (AMR). Diagnosis is confirmed by “*identifying schistocytes, thrombocytopaenia, and microangiopathic haemolytic anaemia, with acute kidney injury (AKI) being the most common form of end-organ damage*” (AMR).

#### CP-ST (stroke in snakebite)

An audience query sparked the conversation on ischaemic stroke in snakebite cases. Ischaemic strokes in snakebite cases were pointed out as “*multifactorial, often resulting from thromboembolic events or watershed infarctions due to hypotension*” (FMS). Snake venom, containing both procoagulants and anticoagulants, leads to varying symptoms depending on the dominant effect (UB). Some species may exhibit stronger procoagulant effects, increasing ischaemic stroke risk (PKK). In the past three years, “*… institute has had at least seven ischaemic infarcts linked to snakebites*”, suggesting underreporting (RPC). The use of thrombolytics and anticoagulants in embolic stroke due to vasculotoxic snakebites remains controversial (AMR). Initial thromboembolic events may give way to haemorrhagic complications, making early thrombolysis potentially harmful (JK).

#### CP-CLS (capillary leak syndrome)

The panel highlighted the early recognition and management of capillary leak syndrome as a significant challenge, particularly in haemotoxic snakebites, where it is a leading cause of mortality (JK). Diagnosing this condition and identifying relevant parameters have only recently become clearer (IM). Capillary leak can affect any organ, with pulmonary involvement leading to high mortality even with ventilatory support (JK). “*Seasonal variations in its occurrence*” warrants further study (JK).

There was a debate over how different antivenom brands influence outcomes, especially regarding complications like capillary leak syndrome (JK) suggesting a need for implementation of standardisation of manufacturing and better quality control.^10^ The potential of “*plasmaphaeresis to remove toxins such as phospholipase A2, zinc metalloprotease, and vascular apoptosis-inducing proteins 1 & 2*” (JK) and “*role of steroids … often administered for capillary leak syndrome*” (JK) remains areas needing further investigation.

#### CP-PED (paediatric snakebite management)

There are no considerable changes in management of paediatric bite in case of antivenom however “*smaller volumes of treatment, generally 5–10 ml/kg*” for paediatric patients was suggested (PKK). It was also noted that “*The response of paediatric patients to treatments or interventions can vary somewhat, and even experienced faculty have limited experience with cases involving newborns and very young*” (PKK). Additionally a comment on, “*many snakebites happen in school settings*” was made noting recent events across India including one death in Wayanad, Kerala.^11^, ^12^, ^13^, ^14^, ^15^

#### CP-RD (regional differences in venom and antivenom potency)

Experts noted significant ‘*differences in antivenom potency in clinical practice*’, emphasising the need for region-specific antivenom production in India. Strategic plans, such as expanding venom centres and implementing policy changes at state and district levels, were discussed as critical for improving antivenom quality.^6^

### Theme 3: Debates and controversies in snakebite protocols (DC)

#### DC-TQ (controversies over tourniquet use)

The use of tourniquets in snakebite management remains highly controversial. “*The use of a tourniquet can provide false reassurance to patients and bystanders*” (RPC). Training is essential because “*when prompt medical care is available, using a tourniquet can often do more harm than good*” (FMS). The use of tourniquets, though generally not recommended, was suggested as a tool that can still be considered “*in remote areas where delays in receiving medical attention*” are expected, “*by experts*” to “*prioritise life over limb*” (FMS). The argument that “*tourniquet has some role in delaying envenomation*” (UB, FMS) was contested by members of the panel (KBM, PKK) and the overarching consensus in national and international guidelines, that the use of tourniquets is not recommended, and the overarching consensus in national and international guidelines, that the use of tourniquets is not recommended, was emphasised by the panellists (MA, PKK).^16^ However to date, none of the panellists reported having seen pressure immobilisation applied to patients they have received.

#### DC-HNPV (antivenom use in hump-nosed pit viper (HNPV) bites)

The management of HNPV bites has sparked significant debate, particularly regarding the use of antivenom. The recognition of ASV's ineffectiveness for pit viper bites “*represents a major shift*” in snakebite management, crucial for “*minimising antivenom wastage*” and emphasising the importance of accurate snake species identification (JK,KBM). “*Diverse factors including ancestral origins, microhabitat preferences and prey-predator interactions of HNPV would have influenced its potency and the higher casualty rates observed in Sri Lanka and Karnataka compared to Kerala*” (SD).

#### DC-PLAS (controversies in plasmaphaeresis)

The role of plasmaphaeresis in treating snakebite complications like capillary leak syndrome remains a topic of debate. “*Although the role of plasmaphaeresis in capillary leak syndrome is controversial in studies, we consider it when a patient develops bilateral parotid swelling, periorbital oedema, haemoconcentration, and refractory shock*” (JK). In some centres it was pointed out that, “*plasmaphaeresis has been used when antivenom was ineffective or not indicated, and has shown potential mortality benefits*”^17^ (AMR). However it was pointed out that in regions like “*Southern Maharashtra, Karnataka, and Goa, Hump-Nosed Pit Vipers are causing more severe complications and higher mortality rates, with plasmaphaeresis often proving ineffective for these patients*”^9^ (FMS). The statement was supported by noting that “*there are no methodological studies supporting the use of plasmaphaeresis for snakebite cases*” (IM). Comprehensive studies at the molecular level are needed to assess the factors involved and the effects of plasmaphaeresis before it can be widely adopted (IM).

#### DC-BT (blood transfusion in snakebite)

The use of blood transfusions in snakebite cases, particularly for managing coagulopathy, is another area of contention. “*Blood component therapy is considered the next best option after administering the full dose of antivenom in cases of coagulopathy, but using it without a clear indication can lead to more complications than benefits*” (JT).

#### DC-SI (issues in snake identification)

Accurate snake identification is essential for effective management but remains challenging. Misidentification, particularly with hump-nosed pit vipers, can lead to inappropriate treatment, such as unnecessary use of ASV, which is ineffective for any of the pit viper species (JK, SD).^5^ Remote identification tools like “*WhatsApp and Facebook groups, along with apps like Snakepedia and SARPA (Snake Awareness Rescue and Protection) can aid in this process*” (SD). However, “*even for experts, identifying a snake from ambiguous photographs, from bite marks or sometimes without knowing its location and other details can be challenging*” (SD).

#### DC-COM (communication)

Effective communication was highlighted as crucial in explaining that “*ASV is ineffective for snake bites outside the Big Four species*” (KKP). Misunderstandings can lead to inappropriate treatment, with “*patients or bystanders sometimes having to convince doctors not to administer ASV*” (SD). Additionally, “*confusion arises when treatment plans change after a patient is transferred between departments*” (FMS), highlighting the need for clear, consistent communication to avoid complications and even harm (UB).

### Theme 4: Policy and research (PR)

This theme focuses on the necessary policy changes and future research directions to improve snakebite management. Discussions emphasised the need for standardised protocols, improvements in antivenom quality, and the importance of data analysis and research to guide effective treatment strategies.

#### PR-PC (policy changes)

The WHO's goal to reduce snakebite deaths by 50% by 2030 drives efforts to expand venom centres and allocate funding.^16^ “*State and district-level action plans are crucial*” (MA), for instance taking the Karnataka model-making snakebite cases notifiable.^18^ Concerns about “*antivenom quality, regulations, and issues with distribution to African countries*” were also raised (MA).^19^^,^^20^

#### PR-FR (future research)

It was noted that, despite substantial data collected by many pioneering faculty members, much of it remained unanalysed, which highlights the gaps in snakebite management research (UB). During this period, “*snakebite management was often neglected*,” which has impacted the development of evidence-based protocols (UB). Predicting and preventing capillary leak, potential of plasmaphaeresis in treating complications like capillary leak syndrome (IM), role of neostigmine (UB), analysis of inflammatory markers, such as matrix metalloproteinases (MMPs) (IM), multicentre production and comparison studies of antivenom to develop region-specific antivenom (UB, KBM, JK), and the analysis of data from major snakebite cases tracked over the past 5–6 years in Kerala (SD) were identified as potential areas for future research. Institutional variation in outcomes using various brands of antivenom, emphasised the importance of research addressing efficacy and complications of antivenom (JK, UB).

### Frequency summary

The thematic analysis identified key focus areas discussed during the panel, with frequency counts providing a contextual overview rather than prioritising or ranking themes.

Theme 1: Evolution of snakebite treatment paradigms (ES); focused on the historical evolution of antivenom use (n = 4), advancements in specialised care (n = 2), and the challenges in early diagnosis (n = 3), giving a total frequency of 9.

Theme 2: Clinical and procedural challenges (CP); n = 3 highlighted various aspects, including challenges in snakebite management (n = 15), paediatric snakebite management (n = 2), allergy and anaphylaxis (n = 3), venom-induced coagulopathy (n = 11), thrombotic microangiopathy (n = 4), stroke in snakebite cases (n = 9), capillary leak syndrome (n = 12), and regional differences in venom and antivenom potency (n = 10), with a total frequency of 66.

Theme 3: Debates and controversies (DC) covered key areas of contention, including tourniquet use (n = 14), antivenom for *H. hypnale* (n = 5), controversies in plasmaphaeresis (n = 5), blood transfusion in snakebite (n = 2), issues in snake identification (n = 11), and communication (n = 7), resulting in a total frequency of 44.

Theme 4: Policy and research (PR); n = 2 discussed the importance of policy changes (n = 7) and the need for future research (n = 11), with a total frequency of 18.

While these frequencies provide a snapshot of the panel's emphasis, the interpretative analysis ensured a deeper exploration of the insights shared, enabling a comprehensive understanding of the challenges and opportunities in snakebite management.

### Cross-cutting themes

#### Cross-cutting themes and theme relationships

**Fig. 1:**
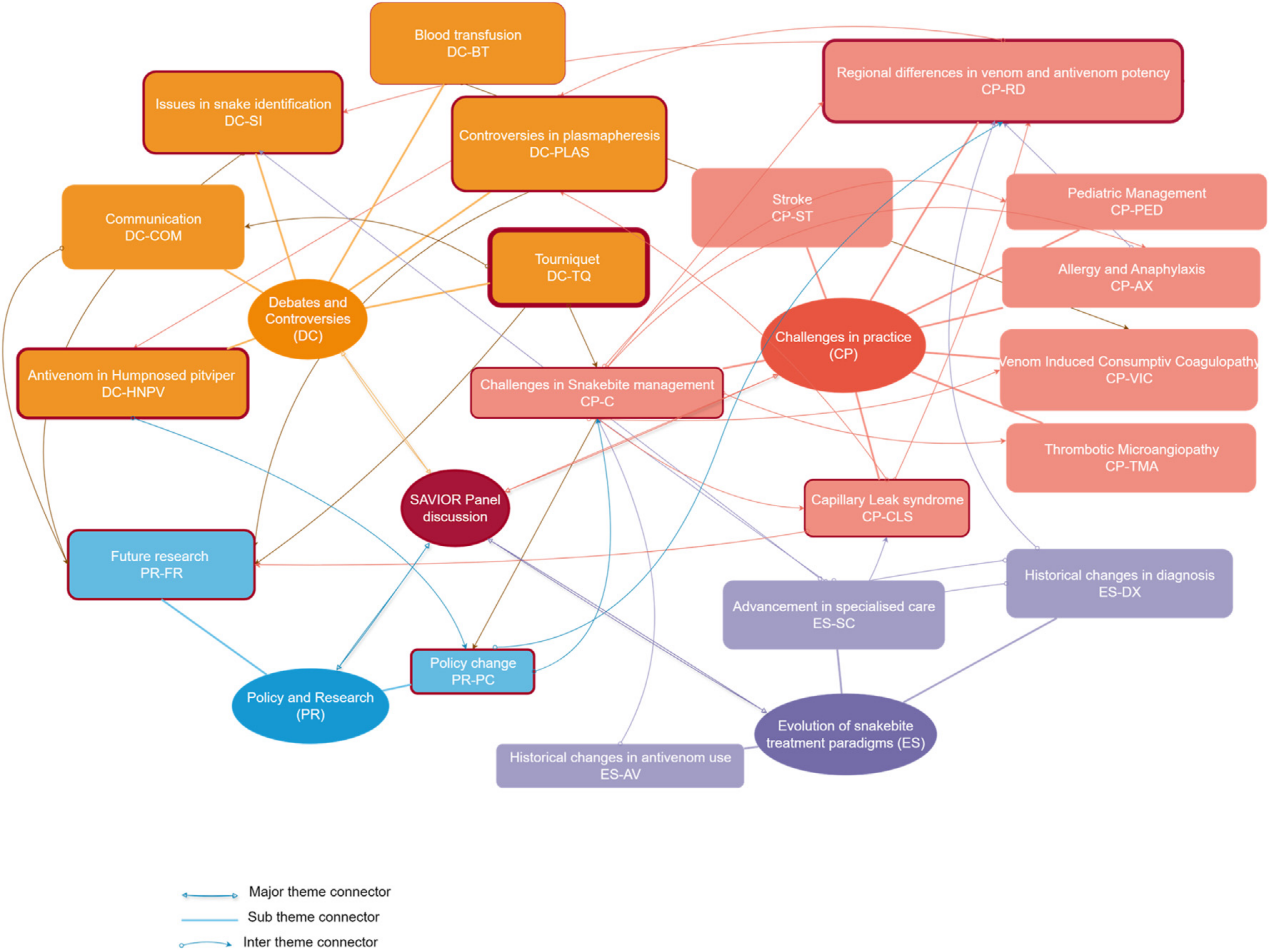
Cross cutting themes. The border thickness of each subtheme is proportional to the number of times the topic was discussed. Each arrow denotes interaction between themes. Thick Borders: Major themes, Thin Borders: Subthemes. Purple Boxes: *Evolution of Snakebite Treatment Paradigms (ES)*, Red Boxes: *Challenges in Practice (CP)*, Orange Boxes: *Debates and Controversies (DC)*, Blue Boxes: *Policy and Research (PR)*. Dark Red Box: *SAVIOUR Panel Discussion* (central connector). Solid Arrows: Major Theme Connectors, Dashed Arrows: Subtheme Connectors, Curved Arrows: Intertheme Connectors. Thicker Arrows: Stronger connections, Thinner Arrows: Weaker connections.

The cross-cutting themes identified in the panel discussion on snakebite management are interconnected and interdependent. Accurate snake species identification, (DC-SI) for instance, is crucial for effective treatment, which in turn relies on high-quality antivenom. Regional differences and variations further complicate treatment strategies, emphasising the need for standardised protocols and guidelines. Data analysis and research are essential for addressing knowledge gaps and developing targeted treatment strategies, which can inform policy changes and improve patient outcomes. Effective collaboration and communication among healthcare providers, researchers, and policymakers are vital for addressing challenges in remote or resource-constrained areas and ensuring that best practices are shared and implemented (Fig. 1).

### Metathemes

1 **Training and strengthening peripheral centres**: Structured programs are crucial for improving healthcare infrastructure at peripheral centres. This includes better notification systems, timely care, and education to reduce delays and improve outcomes (“*Lack of notification and delays in seeking care*”–RPC; “*Inconsistencies in management at referral hospitals*”–JK).
2 **Leveraging digital solutions**: Digital platforms (e.g., Facebook and WhatsApp groups, Snakepedia® app, and SARPA) are valuable for snake identification and data collection. Establishing state-level digital registries is essential for systematic data management and informed decision-making (“*We track major snakebite cases through WhatsApp*”–SD).
3 **Region-specific management protocols**: Protocols must be tailored to local challenges, such as the limitations of existing antivenom in Kerala. Venom toxicity varies with factors like geography and climate, requiring region-specific solutions (“*Regional specificity is important for effective management*”–KBM; “*Venom toxicity varies with location and season*”–UBM).
4 **Antivenom for hump-nosed pit viper (HNPV)**: There's a need for HNPV-specific antivenom due to the ineffectiveness of existing ASV and regional variations in venom potency. HNPV is associated with higher casualty rates and more severe complications (“*ASV is ineffective for pit viper bites*”–KBM; “*HNPV causes higher mortality*”–FMS).

## Discussion

The thematic analysis from the Snakebite Life Support workshop underscores the complex interplay of evolving treatment paradigms, clinical challenges, and region-specific needs in snakebite management. Key themes that evolved in the panel included the importance of strengthening peripheral care through structured training, the development of region-specific protocols, and the use of digital solutions for registry establishment, snake identification, safe transportation, and telesupport. Although scientific advancements are progressing rapidly, existing textbooks struggle to keep pace, and physicians rely on outdated material for clinical practice.^21^^,^^22^ Repeated conditioning has led to acceptance of myths as realities. This growing recognition of the unique challenges in snakebite management has led to its classification as a neglected tropical disease.^23^

### Evolution of snakebite treatment in the region and clinical challenges

The evolution of snakebite treatment in the region reflects a transition from unspecialised care to a more structured approach, characterised by the introduction of antivenom protocols, establishment of specialised care units, and improved diagnostic capabilities. Historical accounts reveal that Indian physicians recognised the need for antivenom as early as the late 18th century.^24^, ^25^, ^26^ Pivotal developments in the 1970s, including haemodialysis, neostigmine, and ventilatory support, significantly reduced mortality from renal failure and neurotoxicity.^27^, ^28^, ^29^, ^30^ Physicians from high volume centres, trialled different doses of antivenom and studied role of heparin in snakebite. They later started noticing inefficiency of the polyvalent antivenom and lack of understanding for pit viper envenomation and even reported seasonal differences in envenomation.^5^^,^^31^, ^32^, ^33^ Despite progress, challenges persist, especially in rural settings where antivenom administration is often delayed due to fears of anaphylaxis and improper referrals. Prophylactic use of subcutaneous adrenaline may mitigate such risks, but its efficacy and safety require robust real-world evaluation.^34^, ^35^, ^36^, ^37^, ^38^, ^39^ The complexity of venom effects, combining procoagulant and anticoagulant properties, complicates treatment, particularly regarding anticoagulants and blood component therapy.

Complications such as CLS remain poorly understood yet contribute to up to 80% of deaths from snakebite in the region.^40^^,^^41^ The complexity of venom-induced coagulopathy, involving both procoagulant and anticoagulant effects, poses challenges for managing thrombotic complications, including stroke and myocardial infarction, Thrombotic microangiopathy (TMA) as well as bleeding disorders further complicate clinical management including transfusion requirements.^8^^,^^17^^,^^42^, ^43^, ^44^, ^45^, ^46^ Addressing these gaps requires a dual approach: improving peripheral care through training and resource allocation while continuing advancements in tertiary care settings to refine snakebite management protocols.

### Need for strengthening the peripheral care settings

Enhancing peripheral care is vital for effective snakebite management. Key priorities include training healthcare workers, providing necessary resources, and empowering peripheral doctors to administer antivenom. In low- and middle-income countries (LMICs), common obstacles such as knowledge gaps, resource shortages (medications, equipment, staffing), and clinician fears hinder effective care.^6^^,^^47^^,^^48^

Many authors have pointed out that doctors and nurses globally often lack the knowledge and confidence to manage snakebites, largely attributed to insufficient and outdated information in medical textbooks, highlighting a critical need for updated education and training.^21^^,^^49^, ^50^, ^51^, ^52^, ^53^ Successful implementation of improved care, requires bridging the gap between theory and practice. Structured clinical training programs, such as the SBLS course, may prove essential for addressing this gap.^50^^,^^51^^,^^53^, ^54^, ^55^ (Supplementary Information S2: Course outline).

### Primary care and referral challenges

Peripheral healthcare systems that have access to antivenom often do not administer it due to fears of complications in most resource limited settings.^54^ The most common complication arising from antivenom administration is an allergic response, which can be effectively managed with proper training, particularly in airway management—an essential skill for both treating allergic reactions and transporting patients with neurotoxic snakebites. Inconsistent clinical practices, such as varying approaches to antivenom or blood product use for *H. hypnale* bites, lead to communication breakdowns and erode trust between healthcare providers and patients. Poor referral systems and inadequate communication between facilities are significant challenges in LMICs lacking structured prehospital care systems.^56^, ^57^, ^58^

### Proposed solutions for improving snakebite outcomes

Majority of snakebites are non-venomous and require observation rather than immediate intervention, while early antivenom administration for venomous bites can prevent complications. Proper triage, timely antivenom use, managing initial complications (like allergies), and ensuring safe referral are crucial. The Snakepedia® app, for instance, connecting 500 regional physicians via WhatsApp, aids in quick snake identification through photographs. Accurate snake identification, combined with a reliable history and training to recognise snakebite syndromes, can reduce healthcare costs.^59^^,^^60^ However, reliance on smartphone-based photography may not be feasible in rural areas due to limited access, and while community education on snake identification is valuable, it should be carefully framed to avoid inadvertently encouraging practices like snake hunting or killing, which can have broader ecological and ethical implications.

Optimal observation policies and referral for venomous bites are often not feasible in peripheral centres due to limited infrastructure and gaps in snakebite care.^48^ Adapting a spoke-and-hub model, used in other medical emergencies, could improve snakebite management. Central hubs would provide advanced care, while peripheral spokes would handle triage, initial treatment, and transport. In unsafe transport scenarios, empowering peripheral healthcare workers to administer antivenom and manage complications is essential. Structured training can alleviate fears of complications and enhance their capacity to act decisively. Clear communication and coordination during transport and arrival at care centres are key to the success of this model, ensuring efficient resource allocation and improved care for snakebite victims.

### Key debates in snakebite treatment: tourniquet use, antivenom efficacy, and plasmaphaeresis

This presents an analysis of the panel discussion, capturing the varying perspectives and debates among the panellists. The disagreements noticed amongst experts in snakebite management itself points towards the need for further research and ensuring the dissemination of guidelines.

#### Pressure immobilisation vs. tourniquet use

The use of pressure bandage with immobilisation (PBI) is often advocated as first aid for snakebite. The real-world effectiveness of PBI has been questioned, as studies show it is often applied incorrectly, leading to inadequate venom containment.^46^, ^47^, ^48^ The evidence supporting PBI largely comes from experimental studies with tagged proteins, which may not fully capture the complexities of real-life envenomation, as venom consists of diverse proteins with varying sizes and kinetics.^61^, ^62^, ^63^, ^64^ Tun Pe et al. observed that non-immobilised, pad-treated systemic cases had venom levels comparable to those without PBI, suggesting that the pad alone is ineffective.^65^^,^^66^ Reports indicate that the complete PBI method, including pressure pad, bandage, and immobilisation, is rarely used as first aid, despite being the standard recommendation.^52^^,^^67^, ^68^, ^69^, ^70^

In contrast, tourniquets, though controversial due to potentially devastating complications like tissue ischaemia and limb loss, is still commonly practiced.^67^^,^^71^, ^72^, ^73^ Most of the science on which snakebite relies on are archaic and scientific re-examination of existing evidence in snakebite first aid is required. Expert opinions in this paper suggest that while tourniquets carry risks, they may be a more practical alternative in regions with limited resources and training, especially when antivenom access is remote (UB, FMS). In Brazil, post *Bothrops jararaca* envenomation, those admitted with a tourniquet in place had significantly higher plasma fibrinogen concentrations than those without a tourniquet cases with envenomation, aligning with the opinion of some panellists (UB, FMS) that it has some role in delaying envenomation.^72^ However, the debate over tourniquets during the panel was heated, with significant opposition due to their inherent risks. The panel's final consensus was not to recommend tourniquets under current evidence, but rather to advocate for rigorous research to evaluate their real-world utility and safety. Efforts should prioritise education and training in proper PBI techniques while exploring practical alternatives for remote and resource-limited settings. The discourse, on the other hand, reflects the broader need for scientific re-evaluation of snakebite first-aid practices to develop evidence-based interventions that are both safe and effective in real-world scenarios.

#### Antivenom efficacy and development of region-specific protocols for effective snakebite management

The effectiveness of antivenoms, particularly polyvalent formulations like the Indian polyvalent antivenom (IPAV), has been a focal point of debate in snakebite management due to its variable efficacy across different snake species and geographic regions.^74^^,^^75^ Standardised treatment protocols for snakebites often fail to account for the regional differences in snake species, venom composition, and healthcare infrastructure. There is a need for region-specific antivenoms, and updated treatment guidelines tailored to the local snake fauna.

Rajasthan, with its desert-adapted snakes, and the North East, where haemotoxic Big Four species are rare, require different management approaches compared to other parts of India.^76^, ^77^, ^78^, ^79^ Physicians' experience and clinical observations both suggest that, the Indian polyvalent antivenom (IPAV) is ineffective against *H. hypnale* venom.^2^^,^^5^^,^^9^^,^^74^^,^^79^, ^80^, ^81^ However, in a recent survey, 43% of respondents indicated they would administer the currently available polyvalent antivenom for snakebites positively identified as *H. hypnale*. They based their decision on “relying on a syndromic approach rather than species identification”, “lacking confidence in accurately identifying the snake”, and “concern about medicolegal implications of withholding antivenom from patients showing signs of envenomation”.^82^ The panel emphasised that administering Indian polyvalent antivenom (IPAV) for *H. hypnale* envenomation could cause more harm than benefit, particularly when the risks of adverse reactions outweigh any potential, albeit unsupported, benefits of IPAV in non-Big Four species (Table 1). Standardised treatment protocols for snakebites often fail to account for the regional differences in snake species, venom composition, and healthcare infrastructure. There is a need for region-specific antivenoms, and updated treatment guidelines tailored to the local snake fauna.

**Table 1:** Text box summarising the consensus and debates warranting future research.

Phase	Consensus	Controversy and debates	Suggested research questions for future exploration
Prehospital	Tourniquet use is not recommended and should be discouraged at the community level as it can cause false reassurance and delay treatment.	In remote areas with delayed access to care, tourniquets may be considered to slow venom spread.	Amongst snakebite victims from (P), does the application of a tourniquet (I), compared to no tourniquet use (C) in the prehospital setting, reduce in-hospital mortality (O)?
	Community education is essential to ensure optimal first aid as well as quick and safe transport of patients to the nearest healthcare centre.		In rural snakebite-endemic communities (P), does a targeted community education programme involving school students and paramedics (I) lead to community behavioural changes and emergency response knowledge (O) within 12 months post-intervention (T)?^a^
	Equipping primary care centres with antivenom and ensuring training courses to empower the peripheral care settings is essential.		In primary care centres in snakebite-endemic regions (P), does equipping centres with antivenom and staff training (I), compared to no specific interventions (C), ensure appropriate antivenom administration and optimal referral (O) within the first 24 h of envenomation (T)?^a^
	Early and optimal referral to higher centres should include early antivenom administration, airway and anaphylaxis management and optimal communication prior to referral.	Concerns about anaphylaxis often prevent necessary antivenom use in peripheral centres.	Amongst healthcare workers at peripheral centres (P), does focused training on anaphylaxis recognition and management during antivenom administration (I), compared to no formal training (C), improve confidence and increase optimal antivenom use (O) over a 12-month period (T)?
In-hospital	IPAV is not useful and should be avoided for non-Big 4 species envenomation including *H. hypnale* envenomation.	There is such considerable regional variation in snakebite envenomation and responses to existing IPAV that there is a need for region-specific protocol guidelines and antivenom.	What are the key factors contributing to regional variations in snakebite envenomation outcomes and treatment responses, and how can these inform the development of region-specific protocol guidelines and antivenom formulations? (Exploratory research)
	Specialised care units can reduce mortality rates with proper critical care.	CLS, VICC, TMA, CVA in snakebite are poorly understood complications. The effectiveness of plasmaphaeresis in these conditions is controversial.	In patients with snakebite induced complications such as CLS, VICC, or TMA (P), does the use of plasmaphaeresis (I) compared to supportive care without plasmaphaeresis (C) in terms of morbidity and mortality (O) within a 6-month follow-up period (T)?
		Responses to treatment in paediatric patients, especially newborns, are less understood.	What are the differences in venom pharmacology and toxicokinetics in paediatric patients? What are the complications specific to this age group, and the factors affecting outcomes including mortality? (Exploratory research)
		The role of anticoagulation or antiplatelets in embolic strokes is controversial.	In snakebite patients with ischaemic strokes following snakebite (P), does early antiplatelet therapy started within 24 h of stroke (I), compared to delayed initiation (C), improve outcomes and reduce complications (O) within 90 days of the stroke (T)?
	Antivenom must be administered with caution due to the risk of anaphylaxis, but full doses are necessary when needed.	The role of premedication prior to antivenom administration is still controversial.	In snakebite patients requiring antivenom (P), does prophylactic low-dose adrenaline (I), compared to no treatment (C), reduce the incidence of anaphylaxis (O), within 6 h of treatment (T)? In snakebite patients with complete venom-induced coagulopathy at presentation (P), does the use of 20 vials (I), compared to 10 vials antivenom (C), reduce unnecessary blood transfusions and complications and aid in early restoration of coagulation factors (O) within the first 48 h of treatment (T)?
	Blood component therapy should be used only a. if indicated, after full dose antivenom administration (when indicated) and b. if there is no adequate improvement in antivenom or with ongoing bleeding manifestations.	Timing and indications of blood component therapy is unclear in snakebite victims.	In snakebite patients with venom-induced coagulopathy (P), does the use of fresh frozen plasma within 6 h of antivenom (I), compared to existing practices (C), reduce complications (O) during hospital stay (T)?
	Accurate snake identification can be beneficial in treatment, especially distinguishing venomous from non-venomous bites and ‘Big four’ from non-big four species. Use of digital platforms like Whatsapp and online apps may be used when feasible for snake identification as well as dissemination of information.		In snakebite-endemic communities (P), how does implementing the use of a community-based WhatsApp group for real-time snake identification and teleconsultation (I), compared to no intervention (C), impact appropriateness of treatment, particularly in guiding antivenom use for Big Four versus non-Big Four species (O) over a 12-month period (T)?^a^
	Clear and uniform communication about ASV's ineffectiveness for non-Big Four species is crucial.	There is lack of clarity in the communication especially with regards to antivenom administration in non-‘Big Four’ species.	In snakebite-endemic communities (P), does the development and implementation of the use of a standard patient decision aid (I) compared to usual care (C) improve patient satisfaction and compliance (O) during the in-hospital stay (T) for patients with snakebites?
Policy	National guidelines need to incorporate standardised snakebite treatment protocols to be disseminated and implemented across the country.		Amongst healthcare workers treating snakebite cases (P), does the implementation of standardised snakebite management protocols (I), compared to no protocols (C), improve patient outcomes and reduce complications (O) over a 6-month period (T)?
	There is a need for stringent regulation of antivenom quality and ensuring effective availability at treatment centres all over India.		

Note: CVA: Cerebrovascular accident; CLS: Capillary leak syndrome; VICC: Venom-induced consumptive coagulopathy; TMA: Thrombotic microangiopathy; IPAV: Indian polyvalent antivenom; ASV: anti-snake venom.

a Eventhough there is expert consensus, further research could help solidify the evidence base and explore potential gaps in the current understanding.

The Australian experience highlights a shift from using monovalent antivenoms guided by unreliable snake venom detection kits to focussing on diagnosing envenomation and administering polyvalent antivenom or two key monovalent antivenoms (brown and tiger snake) based on geography and clinical presentation. This approach has simplified antivenom use, reduced errors, and minimise risks associated with incorrect antivenom administration, highlighting the importance of diagnostic clarity and pragmatic treatment strategies.^83^ Syndromic management, supported by region-specific clinical guidelines, remains the most pragmatic approach until more effective diagnostic and treatment options become available in the region.^84^ The inconsistent adherence to snakebite management protocols across India highlights the need for standardised yet adaptable guidelines that cater to regional specificities, ensuring effective, contextually appropriate care that enhances patient outcomes and optimises resource use.

#### Plasmaphaeresis

Theoretically, therapeutic plasma exchange (TPE) should facilitate the removal of venom toxins from the intravascular compartment, potentially redistributing them from extravascular spaces for subsequent elimination. While early case reports and studies indicate its utility in managing haematologic complications, robust evidence remains sparse.^.^^85^^,^^86^ A retrospective study of 37 snakebite patients treated with TPE showed significant laboratory improvements and limb salvage without complications, supporting its use in complex cases.^87^ However, a systematic review by Noutsos and colleagues found no definitive evidence for TPE's benefit in TMA-associated acute kidney injury (AKI), despite a TMA prevalence of 5.4% in *H. hypnale* bites and 10–15% in Australian elapid envenomations.^88^ The American Society for Apheresis (ASFA) classifies TPE for snakebite envenomation as a Category III intervention, indicating that its optimal role in therapy remains unestablished and decisions should be made on a case-by-case basis.^89^ Plasmaphaeresis, while promising for complications like VICC and thrombotic microangiopathy (TMA), lacks robust evidence from randomised controlled trials.^17^^,^^85^, ^86^, ^87^, ^88^ The panel identified this as a priority for future research to determine its optimal timing, efficacy before integration into treatment protocols, as outlined in Table 1, and emphasised the need for interim observational studies to guide current practice.

#### Bridging knowledge gaps: future research and policy directions in snakebite care

To bridge the gaps in snakebite management and build on existing evidence, targeted research, and policy development are essential in several key areas. Establishing structured training platforms is critical to enhancing the skills of healthcare providers, particularly in peripheral care centres. Strengthening these centres, improving the prehospital care system, optimising referral processes, including notifications and patient reception, and ensuring mandatory reporting of this neglected tropical disease are recognised as necessary steps to improve patient outcomes.^90^ On November 27, 2024, India's Ministry of Health and Family Welfare (MoHFW) has urged all states to make snakebite cases “notifiable disease”.^91^

The standard treatment guidelines (STGs) for snakebite management was published in 2017, following the 2nd edition of WHO treatment guidelines in 2016. The National Action Plan for Prevention and Control of Snakebite Envenoming in India was launched on March 12, 2024.^89^ However there is a clear gap in the dissemination of national action policies and guidelines within the healthcare community, as evidenced by expert commentary. This highlights a pressing need for implementation research to facilitate the integration of evidence-based guidelines and policies, particularly in the areas of first aid practices and the development of optimal referral policies to ensure timely and effective treatment in snakebite management. Recently, India's Department of Health Research has identified snakebite management and research as a national health research priority, developing a district-level integrated patient-centric emergency care model in which time-sensitive emergencies, including snake bite is being envisaged.^92^

There is a pressing need for newer, more effective antivenoms, along with better diagnostic tests, particularly for managing envenomations by species like the *H. hypnale*.^75^ This highlights the importance of regional specificity in antivenom development, given that venom composition can vary significantly due to factors such as geographical location, seasonal changes, and even the snake's hibernation patterns. Table 1 summarises consensus statements, controversies, and research questions outlining the research priorities, offering a roadmap to translate expert opinions into actionable strategies for guidelines and policy.

Further research should also focus on addressing complications and developing treatment guidelines for special conditions especially capillary leak syndrome and VICC.^40^^,^^93^

The integration of digital platforms for snake identification, the establishment of state-maintained registries, and the creation of common platforms for interaction and audit are crucial for ensuring standardised care and improving patient outcomes.^94^ Accurate snake species identification and the production of region-specific antivenoms are critical steps in reducing the impact of snakebites in high-incidence areas.

Although the number of experts involved in this study was limited, the panellists' combined experience of over 300 years in managing snakebite cases adds substantial credibility to the findings. Their diverse experience across various healthcare settings in India provides valuable insights, and we believe this model can serve as a template for future studies. However, the small sample size and reliance on expert opinion in the limited time frame of 90 min, may not fully capture the range of perspectives, particularly from practitioners in rural or under-resourced settings.

Another limitation of this study is the potential contextual bias introduced by the setting in which the data were collected, as the themes may have been influenced by the structured nature of the SBLS workshop. The focus on strengthening peripheral care through training could have overshadowed other critical aspects of snakebite management that were not as extensively discussed during the workshop. Additionally, while expert opinion is valuable, it may not represent the views of all practitioners, particularly in underserved areas. The under-representation of peripheral healthcare providers in the panel limits the study's ability to fully capture the unique challenges faced by practitioners in primary health centres and rural hospitals, despite efforts to incorporate audience queries into the discussion.

Another potential limitation is that while this study focuses predominantly on *H. hypnale* due to its regional relevance and clinical significance, this emphasis may limit the generalisability of the findings to other geographic regions with different snake species, healthcare systems, and envenomation challenges, including Elapidae bites such as kraits and cobras. While thematic analysis provided a systematic framework to interpret the data, we acknowledge that the structured format may have influenced the prioritisation of certain themes over others. This format may not capture the same level of inter-participant dynamics as focus groups or in-depth interviews. Furthermore, the presence of an audience may have influenced the panellists' responses. Despite efforts to maintain reflexivity, the familiarity among panellists and researchers could have introduced unconscious biases during discussions and analysis. We recommend that subsequent studies incorporate complementary qualitative methods, such as focus groups or in-depth interviews, to enrich and expand upon these findings. Nonetheless, this Health Policy is quite important in bringing out the actual issues in snakebite management amongst physicians, the challenges and controversies that persist, offering a pertinent starting point for future research.

## Conclusion

This Health Policy highlights the complex and multifaceted nature of snakebite management in India, emphasising the need for region-specific protocols, enhanced peripheral care, and ongoing research. The discussion reveals the perceived limitations of current antivenoms, the variability in clinical practices, and the importance of accurate snake identification (Table 1). To address these challenges, there is a pressing need for tailored training programs, improved dissemination and implementation of guidelines,^95^ and the development of region-specific antivenoms and protocols, in non-Big Four snakes including *H. hypnale* to strengthen snakebite care in diverse geographic settings. The integration of digital platforms and the establishment of comprehensive data registries will further support standardised care and improve patient outcomes. The insights gained from the panel discussion serves as a foundational framework for further enquiry and policy initiatives that can be better aligned to bridge existing gaps and advance snakebite management in India.

## Contributors

Conceptualisation: SVA; Methodology: SVA; Investigation: AVG, AS, VC, DM; Data Capture: AVG; Formal data analysis: SVA, AS, DM; Data interpretation: SVA, AS; Writing–original draft: SVA; Writing–editing: DM, PKK, UV, FMS, MA, JT, IM, KBM, PCR; Writing–review, critical revision: KS, PKM, VKS, BUP, PCR, SD, JK, AMR; Supervision: BUP, PKK, SVA; Funding acquisition: SVA.

During the preparation of this work, OpenAI's GPT-4.0 was used to assist in generating additional codes during the transcript analysis process. These codes were manually reviewed and assigned by AVG and SVA, then verified by AS. Additionally, OpenAI's GPT-4.0 was used to assist with language refinement, content organisation, and drafting supplementary sections. The authors carefully reviewed and edited all outputs as needed and take full responsibility for the content and integrity of the publication.

## Data sharing statement

The corresponding author would consider sharing the data with others on reasonable request. All the authors involved in the study have access to the data collected as part of this manuscript.

## Declaration of interests

The workshop was funded by the Indian Council for Medical Research (ICMR) through the extramural grant for clinical training of healthcare workers (Grant No. 3/1/3/Clinical training/HRD/2023) and partially funded by the ICMR HISS (Grant No. 5/4/8-21/2021-NCD-II) provided to SVA. The funding sources had no role in the study design, data collection, analysis, interpretation of the data, writing of the manuscript, or the decision to submit it for publication. The authors were not precluded from accessing any data in the study, and they take full responsibility for the content and decision to submit for publication. No authors have been paid by any pharmaceutical company or other agency to write this article. We declare no other competing interests.

## References

[1] Suraweera W., Warrell D., Whitaker R. (2020). Trends in snakebite deaths in India from 2000 to 2019 in a nationally representative mortality study. ELife.

[2] Shivanthan M.C., Yudhishdran J., Navinan R., Rajapakse S. (2014). Hump-nosed viper bite: an important but under-recognized cause of systemic envenoming. J Venom Anim Toxins Incl Trop Dis.

[3] Snake Bite Life Support | SBLS Snake bite workshop | Wilderness medicine | Jubilee Mission Medical College and Research Institute, Thrissur, Kerala, India [Internet]. SBLS. https://www.snakebitelifesupport.com.

[4] Braun V., Clarke V., Hayfield N., Davey L., Jenkinson E., Bager-Charleson S., McBeath A. (2022). Supporting Research in Counselling and Psychotherapy: Qualitative, Quantitative, and Mixed Methods Research.

[5] Joseph J.K., Simpson I.D., Menon N.C.S. (2007). First authenticated cases of life-threatening envenoming by the hump-nosed pit viper (Hypnale hypnale) in India. Trans R Soc Trop Med Hyg.

[6] Gajbhiye R.K., Munshi H., Bawaskar H.S. (2023). National programme for prevention & control of snakebite in India: key challenges & recommendations. Indian J Med Res.

[7] Bawaskar H.S., Bawaskar P.H., Punde D.P., Inamdar M.K., Dongare R.B., Bhoite R.R. (2008). Profile of snakebite envenoming in rural Maharashtra, India. J Assoc Physicians India.

[8] Maduwage K., Isbister G.K. (2014). Current treatment for venom-induced consumption coagulopathy resulting from snakebite. PLoS Negl Trop Dis.

[9] Sirur F.M., Balakrishnan J.M., Lath V. (2022). Hump-nosed pit viper envenomation in Western Coastal India: a case series. Wilderness Environ Med.

[10] Guidelines for the production, control and regulation of snake antivenom immunoglobulins, Annex 5, TRS No 1004 [Internet]. https://www.who.int/publications/m/item/snake-antivenom-immunoglobulins-annex-5-trs-no-1004.

[11] Praveena S.A. (2023). 12-year-old student suffers snakebite at school's loo in Tamil Nadu [Internet]. The New Indian Express. https://www.newindianexpress.com/states/tamil-nadu/2023/Jul/11/12-year-old-student-suffers-snakebite-at-schools-loo-in-tamil-nadu-2593484.html.

[12] Kerala news: girl, 10, dies of snakebite in class in Wayanad, school allegedly ignored injury [Internet]. https://www.ndtv.com/kerala-news/kerala-girl-10-dies-of-snakebite-in-class-in-wayanad-school-allegedly-ignored-injury-2136264.

[13] Staff Reporter (2019). Another snakebite case in Wayanad school [Internet]. The Hindu. https://www.thehindu.com/news/national/kerala/student-hospitalised-snakebite-suspected/article30333323.ece.

[14] Telangana Today (2024). Telangana: four students bitten by snake in school [Internet]. Telangana Today. https://telanganatoday.com/telangana-four-students-bitten-by-snake-in-school.

[15] (2023). Snake bites school student in AMU hostel, critical [Internet]. The Times of India. https://timesofindia.indiatimes.com/city/agra/snake-bites-school-student-in-amu-hostel-critical/articleshow/104146850.cms.

[16] National action plan for prevention and control of snakebite envenoming (NAP-SE) in India by 2030 (Draft) [Internet]. https://ncdc.mohfw.gov.in/WriteReadData/l892s/67801163831702665466.pdf.

[17] Mohan G., Guduri P.R., Shastry S., Kandasamy D. (2019). Thrombotic microangiopathy in hematotoxic snakebites and its impact on the prognosis: an entity often overlooked. J Thromb Thrombolysis.

[18] The Hindu Bureau (2024). Snakebite cases and deaths now notifiable in Karnataka [Internet]. The Hindu. https://www.thehindu.com/news/national/karnataka/snakebite-cases-and-deaths-now-notifiable-in-karnataka/article67863513.ece.

[19] Malesi T. (2023). ‘Ineffective’ India-made antivenoms recalled in Kenya, country faces snakebite crisis [Internet]. Down To Earth. https://www.downtoearth.org.in/africa/-ineffective-india-made-antivenoms-recalled-in-kenya-country-faces-snakebite-crisis-89125.

[20] Dalhat M.M., Potet J., Mohammed A., Chotun N., Tesfahunei H.A., Habib A.G. (2023). Availability, accessibility and use of antivenom for snakebite envenomation in Africa with proposed strategies to overcome the limitations. Toxicon X.

[21] Afroz A., Siddiquea B.N., Shetty A.N., Jackson T.N.W., Watt A.D. (2023). Assessing knowledge and awareness regarding snakebite and management of snakebite envenoming in healthcare workers and the general population: a systematic review and meta-analysis. PLoS Negl Trop Dis.

[22] Chakma J.K., Menon J.C., Dhaliwal R.S. (2020). White paper on venomous snakebite in India. Indian J Med Res.

[23] Chippaux J.P. (2017). Snakebite envenomation turns again into a neglected tropical disease!. J Venom Anim Toxins Incl Trop Dis.

[24] Fayrer J. (1870). The thanatophidia of India: deaths by snake-bite in the Bengal presidency during 1869. Indian Med Gaz.

[25] Hankin E.H. (1896). A supply of snake venom antitoxin for India. Indian Med Gaz.

[26] (1888). Snakes. Halls J Health.

[27] George Renal outcomes among snake-envenomed patients with acute kidney injury in southern India [Internet]. http://www.nmji.in/article.asp?issn=0970-258X;year=2019;volume=32;issue=1;spage=5;epage=8;aulast=George.

[28] Naphade R.W., Shetti R.N. (1977). Use of neostigmine after snake bite. Br J Anaesth.

[29] Banerjee R.N., Sahni A.L., Chacko K.A., Vijay K. (1972). Neostigmine in the treatment of Elapidae bites. J Assoc Physicians India.

[30] Kumar S., Usgaonkar R.S. (1968). Myasthenia gravis--like picture resulting from snake bite. J Indian Med Assoc.

[31] Paul V., Prahlad K.A., Earali J., Francis S., Lewis F. (2003). Trial of heparin in viper bites. J Assoc Physicians India.

[32] Paul V., Pratibha S., Prahlad K., Earali J., Francis S., Lewis F. (2004). High-dose anti-snake venom versus low-dose anti-snake venom in the treatment of poisonous snake bites — a critical study. J Assoc Physicians India.

[33] Kumar P.D. (1994). Snake season. Am J Med.

[34] Premawardhena A.P., de Silva C.E., Fonseka M.M.D., Gunatilake S.B., de Silva H.J. (1999). Low dose subcutaneous adrenaline to prevent acute adverse reactions to antivenom serum in people bitten by snakes: randomised, placebo controlled trial. BMJ.

[35] Dassanayake A.S., Karunanayake P., Kasturiratne K.T.A.A. (2002). Safety of subcutaneous adrenaline as prophylaxis against acute adverse reactions to anti-venom serum in snakebite. Ceylon Med J.

[36] de Silva H.A., Pathmeswaran A., Ranasinha C.D. (2011). Low-dose adrenaline, promethazine, and hydrocortisone in the prevention of acute adverse reactions to antivenom following snakebite: a randomised, double-blind, placebo-controlled trial. PLoS Med.

[37] Gawarammana I.B., Kularatne S.A.M., Dissanayake W.P., Kumarasiri R.P.V., Senanayake N., Ariyasena H. (2004). Parallel infusion of hydrocortisone +/− chlorpheniramine bolus injection to prevent acute adverse reactions to antivenom for snakebites. Med J Aust.

[38] Feng J., Wu Z., Yu Q. (2022). Hydrocortisone for preventing adverse drug reactions to snake antivenom: a meta-analysis. Emerg Med Int.

[39] Horowitz B.Z., Jadallah S., Derlet R.W. (1996). Fatal intracranial bleeding associated with prehospital use of epinephrine. Ann Emerg Med.

[40] Udayabhaskaran V., Arun Thomas E.T., Shaji B. (2017). Capillary leak syndrome following snakebite envenomation. Indian J Crit Care Med.

[41] Kumar K.S., Narayanan S., Udayabhaskaran V., Thulaseedharan N.K. (2018). Clinical and epidemiologic profile and predictors of outcome of poisonous snake bites - an analysis of 1,500 cases from a tertiary care center in Malabar, North Kerala, India. Int J Gen Med.

[42] Isbister G.K., Buckley N.A., Page C.B. (2013). A randomized controlled trial of fresh frozen plasma for treating venom-induced consumption coagulopathy in cases of Australian snakebite (ASP-18). J Thromb Haemost.

[43] Holla S.K., Rao H.A., Shenoy D., Boloor A., Boyanagari M. (2018). The role of fresh frozen plasma in reducing the volume of anti-snake venom in snakebite envenomation. Trop Doct.

[44] Ranawaka U.K. (2024). Bites and stings: exotic causes of stroke in Asia. Cerebrovasc Dis Extra.

[45] Liblik K., Byun J., Saldarriaga C. (2022). Snakebite envenomation and heart: systematic review. Curr Probl Cardiol.

[46] Gn Y.M., Ponnusamy A., Thimma V. Snakebite induced thrombotic microangiopathy leading to renal cortical necrosis. https://onlinelibrary.wiley.com/doi/10.1155/2017/1348749.

[47] Habib A.G. (2013). Public health aspects of snakebite care in West Africa: perspectives from Nigeria. J Venom Anim Toxins Incl Trop Dis.

[48] Bhaumik S., Norton R., Jagnoor J. (2023). Structural capacity and continuum of snakebite care in the primary health care system in India: a cross-sectional assessment. BMC Prim Care.

[49] Menon J.C., Joseph J.K., Whitaker R.E. (2017). Venomous snake bite in India - Why do 50,000 Indians die every year?. J Assoc Physicians India.

[50] Simpson I.D. (2008). A study of the current knowledge base in treating snake bite amongst doctors in the high-risk countries of India and Pakistan: does snake bite treatment training reflect local requirements?. Trans R Soc Trop Med Hyg.

[51] Taieb F., Dub T., Madec Y. (2018). Knowledge, attitude and practices of snakebite management amongst health workers in Cameroon: need for continuous training and capacity building. PLoS Negl Trop Dis.

[52] Mahmood M.A., Halliday D., Cumming R. (2019). Inadequate knowledge about snakebite envenoming symptoms and application of harmful first aid methods in the community in high snakebite incidence areas of Myanmar. PLoS Negl Trop Dis.

[53] Michael G.C., Bala A.A., Mohammed M. (2022). Snakebite knowledge assessment and training of healthcare professionals in Asia, Africa, and the Middle East: a review. Toxicon X.

[54] Barnes K., Ngari C., Parkurito S. (2021). Delays, fears and training needs: perspectives of health workers on clinical management of snakebite revealed by a qualitative study in Kitui County, Kenya. Toxicon X.

[55] Malik A.S., Chatterjee K. (2020). Awareness of Indian medical practitioners about snakebite and its management – Is there a need to re-evaluate medical training?. Med J Dr DY Patil University.

[56] Strand E., Murta F., Tupetz A. (2022). Perspectives on snakebite envenoming care needs across different sociocultural contexts and health systems: a comparative qualitative analysis among US and Brazilian health providers. Toxicon X.

[57] Roy N., Murlidhar V., Chowdhury R. (2010). Where there are no emergency medical services-prehospital care for the injured in Mumbai, India. Prehosp Disaster Med.

[58] Delaney P.G., Moussally J., Wachira B.W. (2024). Future directions for emergency medical services development in low- and middle-income countries. Surgery.

[59] Bolon I., Durso A.M., Botero Mesa S. (2020). Identifying the snake: first scoping review on practices of communities and healthcare providers confronted with snakebite across the world. PLoS One.

[60] Salim A., Williams J., Abdel Wahab S. (2023). Identifying key factors contributing to treatment costs for snakebite envenoming in private tertiary healthcare settings in Tamil Nadu, India. PLoS Negl Trop Dis.

[61] Anker R.L., Loiselle D.S., Straffon W.G., Anker K.M. (1982). Retarding the uptake of “mock venom” in humans: comparison of three first-aid treatments. Med J Aust.

[62] Anker R.L., Straffon W.G., Loiselle D.S., Anker K.M. (1983). Snakebite. Comparison of three methods designed to delay uptake of “mock venom”. Aust Fam Physician.

[63] Tun-Pe, Muang-Muang-Thwin, Myint-Myint-Than, Aye-Aye-Myint, Kyaw-Myintl, Thein Than (1994). The efficacy of compression immobilization technique in retarding spread of radio-labeled Russell's viper venom in rhesus monkeys and “mock venom” NaI131 in human volunteers. Southeast Asian J Trop Med Public Health.

[64] Howarth D.M., Southee A.E., Whyte I.M. (1994). Lymphatic flow rates and first-aid in simulated peripheral snake or spider envenomation. Med J Aust.

[65] Tun-Pe, Tin-Nu-Swe, Myint-Lwin, Warrell D.A., Than-Win (1987). The efficacy of tourniquets as a first-aid measure for Russell's viper bites in Burma. Trans R Soc Trop Med Hyg.

[66] Tun-Pe, Aye-Aye-Myint, Khin-Ei-Han, Thi-Ha, Tin-Nu-Swe (1995). Local compression pads as a first-aid measure for victims of bites by Russell's viper (Daboia russelii siamensis) in Myanmar. Trans R Soc Trop Med Hyg.

[67] White J., Alfred S., Bates D. (2019). Twelve month prospective study of snakebite in a major teaching hospital in Mandalay, Myanmar; Myanmar Snakebite Project (MSP). Toxicon X.

[68] Norris R.L., Ngo J., Nolan K., Hooker G. (2005). Physicians and lay people are unable to apply pressure immobilization properly in a simulated snakebite scenario. Wilderness Environ Med.

[69] Canale E., Isbister G.K., Currie B.J. (2009). Investigating pressure bandaging for snakebite in a simulated setting: bandage type, training and the effect of transport. Emerg Med Australas.

[70] Simpson I.D., Tanwar P.D., Andrade C., Kochar D.K., Norris R.L. (2008). The Ebbinghaus retention curve: training does not increase the ability to apply pressure immobilisation in simulated snake bite--implications for snake bite first aid in the developing world. Trans R Soc Trop Med Hyg.

[71] Bhat R.N. (1974). Viperine snake bite poisoning in Jammu. J Indian Med Assoc.

[72] França F.O.S., Barbaro K.C., Fan H.W. (2003). Envenoming by Bothrops jararaca in Brazil: association between venom antigenaemia and severity at admission to hospital. Trans R Soc Trop Med Hyg.

[73] Melit R.J., Abraham S.V., Radhakrishnan S. (2021). Retrospective review of case records of snakebite presenting to a single tertiary care centre over a 5-year period. Natl Med J India.

[74] Soopairin S., Patikorn C., Taychakhoonavudh S. (2023). Antivenom preclinical efficacy testing against Asian snakes and their availability in Asia: a systematic review. PLoS One.

[75] Gopal G., Selvaraj H., Venkataramanan S.K. (2024). Systematic review and meta-analysis on the efficacy of Indian polyvalent antivenom against the Indian snakes of clinical significance. Arch Toxicol.

[76] Kakati H., Giri S., Patra A. (2023). A retrospective analysis of epidemiology, clinical features of envenomation, and in-patient management of snakebites in a model secondary hospital of Assam, North-east India. Toxicon.

[77] Laxme R.R.S., Khochare S., de Souza H.F. (2019). Beyond the ‘big four’: venom profiling of the medically important yet neglected Indian snakes reveals disturbing antivenom deficiencies. PLoS Negl Trop Dis.

[78] Laxme R.R.S., Khochare S., Attarde S. (2021). Biogeographic venom variation in Russell's viper (Daboia russelii) and the preclinical inefficacy of antivenom therapy in snakebite hotspots. PLoS Negl Trop Dis.

[79] Khochare S., Senji Laxme R.R., Jaikumar P. (2023). Fangs in the Ghats: preclinical insights into the medical importance of pit vipers from the Western Ghats. Int J Mol Sci.

[80] Ariaratnam C.A., Thuraisingam V., Kularatne S.A.M. (2008). Frequent and potentially fatal envenoming by hump-nosed pit vipers (Hypnale hypnale and H. nepa) in Sri Lanka: lack of effective antivenom. Trans R Soc Trop Med Hyg.

[81] Namal Rathnayaka R.M.M.K., Ranathunga P.E.A.N., Kularatne S.A.M. (2020). Venom-induced consumption coagulopathy following hump-nosed pit viper (Genus: Hypnale) envenoming in Sri Lanka: uncertain efficacy of fresh frozen plasma. Wilderness Environ Med.

[82] Abraham S.V., Paul S., Mathew D., Rajeev P.C., Paul M.V., Davis C. (2025). Challenges in snakebite management in India: insights from a physician survey with special focus on Kerala and treatment of bites by hump-nosed pit vipers (hypnale spp.). Wilderness Environ Med.

[83] Isbister G.K. (2022). Antivenom availability, delays and use in Australia. Toxicon X.

[84] Abraham S.V. (2018). Snake bite in India: a few matters to note. Toxicol Rep.

[85] Mohan G., Guduri P.R., Shastry S. (2019). Role of therapeutic plasma exchange in snake bite associated thrombotic microangiopathy-A case report with review of literature. J Clin Apher.

[86] Rathnayaka R.M.M.K.N., Nishanthi Ranathunga P.E.A., Kularatne S.A.M., Sugathadasa K. (2022). Therapeutic plasma exchange for venom-induced thrombotic microangiopathy following hump-nosed pit viper (Genus: Hypnale) bites: a prospective observational study. Wilderness Environ Med.

[87] Zengin S., Yilmaz M., Al B. (2013). Plasma exchange as a complementary approach to snake bite treatment: an academic emergency department's experiences. Transfus Apher Sci.

[88] Noutsos T., Currie B.J., Lek R.A., Isbister G.K. (2020). Snakebite associated thrombotic microangiopathy: a systematic review of clinical features, outcomes, and evidence for interventions including plasmapheresis. PLoS Negl Trop Dis.

[89] Connelly-Smith L., Alquist C.R., Aqui N.A. (2023). Guidelines on the use of therapeutic apheresis in clinical practice – evidence-based approach from the Writing Committee of the American Society for Apheresis: the Ninth Special Issue. J Clin Apher.

[90] Union health Ministry launches national action plan for prevention and control of snakebite envenoming in India – An initiative to halve the snakebite deaths by 2030 through ‘one health’ approach [Internet]. https://pib.gov.in/pib.gov.in/Pressreleaseshare.aspx?PRID=2013803.

[91] Bajeli-Datt K. (2024). Snakebite declared ‘notifiable disease’, Centre to meet WHO target to reduce global deaths by 2030 [Internet]. The New Indian Express. https://www.newindianexpress.com/nation/2024/Nov/30/snakebite-declared-notifiable-disease-centre-to-meet-who-target-to-reduce-global-deaths.

[92] EOI_Hqaes18042023.pdf [Internet]. https://main.icmr.nic.in/sites/default/files/upload_documents/EOI_Hqaes18042023.pdf.

[93] Abraham S.V., Hakkeem B., Mathew D. (2020). Hematotoxic snakebite victim with trauma: the role of guided transfusion, rotational thromboelastometry, and tranexamic acid. Wilderness Environ Med.

[94] Durso A.M., Bolon I., Kleinhesselink A.R. (2021). Crowdsourcing snake identification with online communities of professional herpetologists and avocational snake enthusiasts. R Soc Open Sci.

[95] Sreekumar A., Abraham S.V., Rajeev P.C. (2025). Educating healthcare workers in snakebite management: a study to determine the effectiveness of the snake bite life support workshop. Toxicon.

