# Supplementary material for: Clinical challenges, controversies, and regional strategies in snakebite care in India
Source: Lancet Reg Health Southeast Asia. 2025 May 15;37:100598. doi: 10.1016/j.lansea.2025.100598 (PMC12145746; doi:10.1016/j.lansea.2025.100598)
Supplement: Supplementary Information S1 [file mmc1.docx]

**Supplementary Information 1**

**The Snake Bite Life Support (SBLS) workshop Panellists' Profile**

Dr. Purushothaman K.K.

- **Title**: Professor of Pediatrics
- **Specialisation**: M.D. (Pediatrics)
- **Institution/Affiliation**: MES Medical College, Perinthalmanna, Government Medical College, Thrissur
- **Years of Experience Managing Snakebite**: 45 years
- **Management Focus**: Extensive experience in snake bites in children and instrumental in establishing primary health care policies.
- **Geographical Focus**: Thrissur, Thiruvananthapuram, Kozhikode, and Kasargod
- **Publications/Research Interests**: Developed Kerala state guidelines and educational videos on snakebite management.

Dr. Indira M.

- **Title**: Professor in General Medicine and Medical Toxicology
- **Specialisation**: M.D. (General Medicine), MSc. (Medical Toxicology)
- **Institution/Affiliation**: Government Medical College, Ernakulam
- **Years of Experience Managing Snakebite**: 20 years
- **Management Focus**: Managed snakebite cases and initiated a WhatsApp group for snake identification and registry
- **Geographical Focus**: Northern and Central Kerala
- **Publications/Research Interests**: Multiple presentations on snakebites at national and international conferences. Guided multiple theses on snakebite management.

Sandeep Das

- **Title**: Herpetologist
- **Specialisation**: Ph.D. in Taxonomy, Ecology & Behaviour
- **Institution/Affiliation**: Department of Zoology, University of Calicut
- **Years of Experience Managing Snakebite**: 14 years
- **Management Focus**: Master Trainer and committee member of Snake Rescue Guidelines, Team member of Snakepedia, SARPA
- **Geographical Focus**: Western Ghats
- **Publications/Research Interests**: Authored over 30 scientific papers. Described over 20 new species of amphibians, lizards, and snakes endemic to the Western Ghats.

Dr. Aboobacker Mohamed Rafi

- **Title**: Associate Professor in Immunohematology & Transfusion Medicine
- **Institution/Affiliation**: Jubilee Mission Medical College & Research Institute, Thrissur
- **Years of Experience Managing Snakebite**: 15 years
- **Management Focus**: Coagulation tests in snakebite cases, pursuing PhD in Coagulopathy in Snakebite, expertise in viscoelastic testing
- **Geographical Focus**: Thrissur, Southeast Asia
- **Publications/Research Interests**: Co-authored multiple papers on coagulation in snakebite victims and currently pursuing a PhD on in vitro assessment of Tranexamic acid in snakebite coagulopathy.

Dr. Joe Thomas K.

- **Title**: Professor in General Medicine
- **Specialisation**: General Medicine
- **Institution/Affiliation**: Jubilee Mission Medical College, Thrissur
- **Years of Experience Managing Snakebite**: 25 years
- **Management Focus**: Focused on delayed complications from snakebites
- **Geographical Focus**: Central Kerala
- **Publications/Research Interests**: Research on guided transfusion and rotational thromboelastometry in haematotoxic snakebite victims.

Dr. Udayabhaskaran V.

- **Title**: Professor in General Medicine
- **Specialisation**: M.D. (General Medicine)
- **Institution/Affiliation**: Malabar Medical College, Kozhikode
- **Years of Experience Managing Snakebite**: 40 years
- **Management Focus**: Focused on capillary leak syndrome in snakebites
- **Geographical Focus**: Northern Kerala
- **Publications/Research Interests**: Published on capillary leak syndrome and the clinical profile of snakebite cases.

Dr. Manu Ayyan

- **Title**: Associate Professor in Emergency Medicine
- **Specialisation**: M.D. (Emergency Medicine), FACEE
- **Institution/Affiliation**: Jawaharlal Institute of Postgraduate Medical Education & Research, Puducherry (JIPMER)
- **Years of Experience Managing Snakebite**: 12 years
- **Management Focus**: Core Committee member for NAPSE, Qualitative research trainer and expert
- **Geographical Focus**: Northern Kerala, Tamil Nadu, Puducherry
- **Publications/Research Interests**: Research on species-specific snakebite management.

Dr. Freston Marc Sirur

- **Title**: Associate Professor of Emergency Medicine
- **Specialisation**: M.D. (Emergency Medicine)
- **Institution/Affiliation**: Manipal Academy of Higher Education
- **Years of Experience Managing Snakebite**: 10 years
- **Management Focus**: Wilderness medicine expert, snake rescuer
- **Geographical Focus**: Thrissur, Thiruvananthapuram, Kozhikode, Kasargod
- **Publications/Research Interests**: Published on snakebite-induced mortality and Hump-Nosed Pit Viper envenomation.

Dr. P C Rajeev

- **Title**: Professor of Emergency Medicine
- **Specialisation**: M.D. (General Medicine), Fellow of the Academic College of Emergency Experts
- **Institution/Affiliation**: Jubilee Mission Medical College & Research Institute, Thrissur
- **Years of Experience Managing Snakebite**: 20 years
- **Management Focus**: Executive member of Aranyakam Nature Foundation, Naturalist
- **Geographical Focus**: Central Kerala
- **Publications/Research Interests**: Environmental conservation and herpetofaunal surveys, with a focus on snakebite management.

Dr. K.B. Mohan

- **Title**: Professor Emeritus in General Medicine
- **Specialisation**: M.D. (General Medicine)
- **Institution/Affiliation**: Daya General Hospital, Thrissur Medical College, Jubilee Mission Medical College
- **Years of Experience Managing Snakebite**: 45 years
- **Management Focus**: Pioneer in snakebite ICU management, focus on pit viper and coagulopathy
- **Geographical Focus**: Northern and Central Kerala
- **Publications/Research Interests**: Focused on snakebite management protocols and the development of snakebite ICUs.

Dr. Jayesh Kumar

- **Title**: Professor in General Medicine
- **Specialisation**: M.D. (General Medicine)
- **Institution/Affiliation**: Government Medical College, Kozhikode
- **Years of Experience Managing Snakebite**: 35 years
- **Management Focus**: Focused on myocarditis following cobra bites
- **Geographical Focus**: Northern and Central Kerala
- **Publications/Research Interests**: Research on clinical features of snakebite-induced capillary leak syndrome and myocarditis following cobra bites.
