# Supplementary material for: Clinical challenges, controversies, and regional strategies in snakebite care in India
Source: Lancet Reg Health Southeast Asia. 2025 May 15;37:100598. doi: 10.1016/j.lansea.2025.100598 (PMC12145746; doi:10.1016/j.lansea.2025.100598)
Supplement: Supplementary Information S2 [file mmc2.docx]

**Supplementary Information 2**

**Coded transcript**

| **Theme** | **Subtheme** | **Code** | **Person**  **(authors*)** | **Comment** | **Frequency of codes** |
| --- | --- | --- | --- | --- | --- |
|  |  |  |  |  |  |
| Evolution of Snakebite Treatment Paradigms (ES) | Historical Changes in Antivenom Use | ES-AV | KBM | "In the mid-1970s, antivenom was limited and typically administered in low doses, with a standard treatment of around 10 vials regardless of the snake species. At that time, it was believed that this dosage was adequate to counteract the venom of the 'big four' species." | 4 |
|  |  |  | KBM | "The serious nature of pit viper bites was fully acknowledged in 2006, leading to a major shift in snakebite management practices." |  |
|  |  |  | KBM | "Recognising that ASV is ineffective for pit viper bites represents a major shift. This understanding has been crucial for minimising antivenom wastage, reducing the need for antivenom, and highlighting the importance of accurate snake species identification." |  |
|  |  |  | FMS | "The identification of snakebite species has become increasingly important, marking a significant shift in management practices." |  |
|  | Advancements in Specialised Care | ES-SC | KBM | "In 1979, a specialised critical care unit for snakebite cases was established at Calicut Medical College in response to high mortality rates from complications like capillary leak syndrome, acute renal injury, and cardiorespiratory and neurological issues. The introduction of haemodialysis and ventilatory support in these units greatly reduced mortality rates." | 2 |
|  |  |  | UB | "The limited number of clinical experts specialising in snakebite management created a significant burden on those in the field. Additionally, there was a lack of local dialysis facilities for treating snakebite cases, so that person was sent to Chennai for training in dialysis." |  |
|  | Historical Changes in Diagnosis | ES-DX | KBM | "Initially thought to be a subspecies of the saw-scaled viper that did not respond to antivenom serum (ASV), the snake was later identified as a Hump-Nosed Pit Viper (HNPV). At that time, there was limited literature available to distinguish between these species." | 3 |
|  |  |  | UB | "Many pioneering faculty members collected substantial amounts of data, but much of it remained unanalysed. During this period, snakebite management was often neglected and limited to certain centres." |  |
|  |  |  | UB | "The limited number of clinical experts specialising in snakebite management created a significant burden on those in the field. Additionally, there was a lack of local dialysis facilities for treating snakebite cases so that person was sent to Chennai for training in dialysis." |  |
|  | | | | | |
| Clinical and Procedural Challenges (CP) | Challenges in Snakebite Management | CP-C | JK | "There is considerable variation in the presentation of individual snakebite cases, often compounded by delays in reaching the hospital after a bite and inconsistencies in management at referral hospitals." | 15 |
|  |  |  | RPC | "One major issue is the lack of notification, which means patients often present without any history of a snakebite, making diagnosis difficult, especially when symptoms are non-specific." |  |
|  |  |  | RPC | "Another challenge is the lack of resuscitation support at peripheral centres." |  |
|  |  |  | RPC | "Delays in seeking emergency care further complicate matters; patients may visit traditional healers, who might send them home without observing warning signs, not considering the possibility of delayed symptoms." |  |
|  |  |  | RPC | "Additionally, the use of a tourniquet can provide false reassurance to patients and bystanders, leading to patients arriving at the hospital without proper immobilisation." |  |
|  |  |  | RPC | "Sometimes, antivenom (ASV) may not be administered even when needed due to concerns about anaphylaxis or confusion about its indications." |  |
|  |  |  | FMS | "Anaphylaxis can occur with any amount of antivenom (ASV). Therefore, the full required dose should be given, and the patient should be monitored before transfer." |  |
|  |  |  | FMS | "Given that most areas are now well-connected by road, which minimises delays in reaching medical care, the use of tourniquets is generally not recommended. However, in remote areas where there may be delays in receiving medical attention, using a tourniquet might be considered if it helps prioritise life over limb." |  |
|  |  |  | PKK | "More snakebites occur at night, and these are often less frequently diagnosed. The challenge is exacerbated by the painless bites of the common krait." |  |
|  |  |  | FMS | "The issue arises when a patient arrives at the emergency department with a bite from an unknown snake species, which turns out to be non-venomous, and presents with local swelling above the tourniquet. The question then is whether antivenom (ASV) should be administered." |  |
|  |  |  | KBM | "In the mid-1970s, antivenom was limited and typically administered in low doses, with a standard treatment of around 10 vials regardless of the snake species. At that time, it was believed that this dosage was adequate to counteract the venom of the 'big four' species." |  |
|  |  |  | JK | "The non-specific nature of snakebite symptoms presents a significant challenge; for example, Cobra bites have been linked to cardiomyopathy, sparking debate over the potential use of beta blockers in treatment." |  |
|  |  |  | FMS | "In Southern Maharashtra, Karnataka, and Goa, Hump-Nosed Pit Vipers (HNPV) are causing more severe complications and higher mortality rates, with plasmapheresis often proving ineffective for these patients. Additionally, venom-induced consumption coagulopathy can lead to systemic manifestations such as stroke, myocardial infarction, and acute kidney injury." |  |
|  |  |  | PKK | "Of course, the main highlight is strengthening the peripheral unit for early stabilisation and administration of ASV. However, by the time they reach tertiary centers, complications have already set in." |  |
|  |  |  | Audience | "All snakebites we refer to the medical college. That's safest for us and the patient. We are ill-equipped to take care of snakebites." |  |
|  | Pediatric Snakebite Management | CP-PED | PKK | "The response of paediatric patients to treatments or interventions can vary somewhat, and even experienced faculty have limited experience with cases involving newborns." | 2 |
|  |  |  | PKK | "Many snakebites happen in school settings, and it is recommended to use smaller volumes of treatment, generally 5-10 ml/kg." |  |
|  | Allergy and Anaphylaxis | CP-AX | RPC | "Sometimes, antivenom (ASV) may not be administered even when needed due to concerns about anaphylaxis or confusion about its indications." | 3 |
|  |  |  | FMS | "Anaphylaxis can occur with any amount of antivenom (ASV). Therefore, the full required dose should be given, and the patient should be monitored before transfer." |  |
|  |  |  | UB | "Administering ASV unnecessarily can lead to more complications, such as anaphylaxis, than any potential benefit to the patient." |  |
|  | Venom-Induced Coagulopathy | CP-VIC | FMS | "Venom-induced consumption coagulopathy can lead to systemic manifestations such as stroke, myocardial infarction, and acute kidney injury." | 11 |
|  |  |  | FMS | "In Southern Maharashtra, Karnataka, and Goa, Hump-Nosed Pit Vipers (HNPV) are causing more severe complications and higher mortality rates, with plasmapheresis often proving ineffective for these patients. Additionally, venom-induced consumption coagulopathy can lead to systemic manifestations such as stroke, myocardial infarction, and acute kidney injury." |  |
|  |  |  | JT | "Steroids are still administered for capillary leak syndrome, often based on cautious optimism.” |  |
|  |  |  | JT | Blood component therapy is considered the next best option after administering the full dose of antivenom in cases of coagulopathy, but using it without a clear indication can lead to more complications than benefits." |  |
|  |  |  | UB | "Since snake venom contains both procoagulants and anticoagulants, the dominant factors determine the presentation of symptoms.” |  |
|  |  |  | UB | "In cases of hemispheric stroke, which is typically a major vessel embolic stroke, is anticoagulation an appropriate treatment option for snake bite cases?" |  |
|  |  |  | PKK | "We need to study the coagulant and anticoagulant properties of each snake species individually. For example, the Malabar pit viper exhibits a stronger procoagulant effect than anticoagulant, leading to a higher likelihood of stroke." |  |
|  |  |  | AMR | "The use of anticoagulation for patients with embolic stroke caused by vasculotoxic snake bites remains controversial." |  |
|  |  |  | JK | "Although initial thromboembolic phenomena may be observed, patients can later develop hemorrhagic issues. Early thrombolysis might worsen the patient's clinical condition." |  |
|  |  |  | RPC | "Strokes in snake bite cases are multifactorial and can result from thromboembolic events or watershed infarctions caused by hypotension." |  |
|  |  |  | SVA | "Over the past three years, there have been seven cases of ischemic infarcts associated with snake bites in Jubilee Mission Medical College, Thrissur." |  |
|  | Thrombotic Microangiopathy (TMA) | CP-TMA | AMR | "Thrombotic microangiopathy (TMA) is not uncommon in snakebite cases in India, often presenting with atypical symptoms. Diagnosis is confirmed by identifying schistocytes, thrombocytopenia, and microangiopathic haemolytic anaemia, with acute kidney injury (AKI) being the most common form of end-organ damage." | 4 |
|  |  |  | AMR | "In some centres, plasmapheresis has been used when antivenom was ineffective or not indicated, and it has shown potential mortality benefits. |  |
|  |  |  | FMS | "In Southern Maharashtra, Karnataka, and Goa, Hump-Nosed Pit Vipers (HNPV) are causing more severe complications and higher mortality rates, with plasmapheresis often proving ineffective for these patients." |  |
|  |  |  | RPC | "Strokes in snake bite cases are multifactorial and can result from thromboembolic events or watershed infarctions caused by hypotension." |  |
|  | Stroke in Snakebite Cases | CP-ST | RPC | "Strokes in snakebite cases are multifactorial and can result from thromboembolic events or watershed infarctions caused by hypotension." | 9 |
|  |  |  | FMS | "In Southern Maharashtra, Karnataka, and Goa, Hump-Nosed Pit Vipers (HNPV) are causing more severe complications and higher mortality rates, with plasmapheresis often proving ineffective for these patients. Additionally, venom-induced consumption coagulopathy can lead to systemic manifestations such as stroke, myocardial infarction, and acute kidney injury." |  |
|  |  |  | UB | "Since snake venom contains both procoagulants and anticoagulants, the dominant factors determine the presentation of symptoms." |  |
|  |  |  | PKK | "We need to study the coagulant and anticoagulant properties of each snake species individually. For example, the Malabar pit viper exhibits a stronger procoagulant effect than anticoagulant, leading to a higher likelihood of stroke." |  |
|  |  |  | AMR | "The use of anticoagulation for patients with embolic stroke caused by vasculotoxic snake bites remains controversial." |  |
|  |  |  | JK | "Although initial thromboembolic phenomena may be observed, patients can later develop haemorrhagic issues. Early thrombolysis might worsen the patient's clinical condition." |  |
|  |  |  | RPC | "Strokes in snake bite cases are multifactorial and can result from thromboembolic events or watershed infarctions caused by hypotension." |  |
|  |  |  | SVA | "Over the past three years, there have been seven cases of ischemic infarcts associated with snake bites in Jubilee Mission Medical College, Thrissur. |  |
|  |  |  | UB | "In cases of hemispheric stroke, which is typically a major vessel embolic stroke, is anticoagulation an appropriate treatment option for snake bite cases?" |  |
|  | Capillary Leak Syndrome (CLS) | CP-CLS | JK | "An increase in the toxicity and complications associated with hemotoxic bites has been observed. Early recognition of capillary leak syndrome, the primary cause of mortality in hemotoxic bites, remains challenging. Despite the use of ASV and supportive care, mortality due to capillary leak is high, even after correcting coagulation parameters." | 12 |
|  |  |  | KBM | "In 1979, a specialised critical care unit for snakebite cases was established at Calicut Medical College in response to high mortality rates from complications like capillary leak syndrome, acute renal injury, and cardiorespiratory and neurological issues. The introduction of haemodialysis and ventilatory support in these units greatly reduced mortality rates." |  |
|  |  |  |  |  |  |
|  |  |  | JK | "Although the role of plasmapheresis in capillary leak syndrome is controversial in studies, we consider it when a patient develops bilateral parotid swelling, periorbital oedema, haemoconcentration, and refractory shock." |  |
|  |  |  | JK | "Capillary leak can occur in any organ, including the lungs, where leakage into the alveoli can result in high mortality, even when the patient is placed on a ventilator. The removal of toxins like Phospholipase A2, Zinc Metalloprotease, and Vascular Apoptosis-Inducing Proteins 1 & 2 (VAIP 1&2) via plasmapheresis shows promise in managing capillary leak syndrome." |  |
|  |  |  | JK | "Cases have demonstrated a seasonal variation in the occurrence of capillary leak, further complicating management strategies." |  |
|  |  |  | JK | "There has been debate about how different brands of antivenom affect outcomes, particularly in relation to complications like capillary leak syndrome. It is essential to examine molecular-level inflammatory markers that contribute to capillary leak." |  |
|  |  |  | IM | "Until recently, diagnosing capillary leak syndrome and determining the relevant parameters were unclear. Predicting and preventing this condition still requires further research and investigation. Currently, there are no methodological studies supporting the use of plasmapheresis for snakebite cases, so it is not yet recommended." |  |
|  |  |  | IM | "Comprehensive studies at the molecular level are needed to analyse the factors involved and the effects of plasmapheresis before it can be considered a viable treatment." |  |
|  |  |  | IM | "Some inflammatory markers, such as matrix metalloproteinases (MMPs), are too small to be removed by plasmapheresis. However, we know that certain markers can activate the complement system, contributing to capillary leak syndrome." |  |
|  |  |  | JT | "Steroids are still administered for capillary leak syndrome, often based on cautious optimism. |  |
|  |  |  | JT | Blood component therapy is considered the next best option after administering the full dose of antivenom in cases of coagulopathy but using it without a clear indication can lead to more complications than benefits." |  |
|  |  |  | RPC | "Strokes in snake bite cases are multifactorial and can result from thromboembolic events or watershed infarctions caused by hypotension." |  |
|  | Regional Differences in Venom and Antivenom Potency | CP-RD | JK | "Recently, there has been an increase in cases involving Hump-Nosed Pit Vipers and Malabar Pit Vipers, in contrast to the more common Russell's Viper cases. Notably, antivenom serum is ineffective against these species." | 10 |
|  |  |  | UB | "Antivenom production involves collecting venom, which can vary due to factors such as geographical location, seasonal changes, hibernation, and captivity. To address this, it is necessary to conduct multicentre production and comparison studies of antivenom to determine if region-specific antivenom should be developed." |  |
|  |  |  | KBM | "Ideally, antivenom should be produced for each individual snake species, and region-specific antivenom should be developed to ensure effective treatment. Regional specificity is important because antivenom may not neutralise every molecule, potentially leading to complications even if it targets the specific snake. Additionally, significant variation in venom toxicity—both in quality and quantity—occurs, with seasonal variations being particularly notable." |  |
|  |  |  | JK | "Institutions are using various brands of antivenom, highlighting the need to compare these brands to assess their efficacy and associated complications." |  |
|  |  |  | JT | "Some centres use liquid antiserum and face storage issues, which require a temperature range of 2-8°C. Inconsistent adherence to these storage requirements can impact efficacy." |  |
|  |  |  | MA | “The strategy involves setting clear directions, timelines, and process indicators, followed by effective monitoring to improve snakebite identification and management. Districts receive direct funds with oversight for training, while state-level initiatives are crucial. For example, Karnataka has made snakebite cases notifiable. These strategic plans are designed to provide clear guidance on what needs to be done over the next decade.” |  |
|  |  |  | MA | "There are concerns about the quality and efficacy of antivenom, as some are even distributed to African countries." |  |
|  |  |  | RPC | "Sometimes, antivenom (ASV) may not be administered even when needed due to concerns about anaphylaxis or confusion about its indications. There is also significant confusion and inconsistency regarding the appropriate dosage of ASV for different snakebites." |  |
|  |  |  | FMS | "In Southern Maharashtra, Karnataka, and Goa, Hump-Nosed Pit Vipers (HNPV) are causing more severe complications and higher mortality rates, with plasmapheresis often proving ineffective for these patients. Additionally, venom-induced consumption coagulopathy can lead to systemic manifestations such as stroke, myocardial infarction, and acute kidney injury." |  |
|  |  |  | SD | "Diverse factors including ancestral origins, microhabitat preferences and prey-predator interactions of HNPV would have influenced its potency and the higher casualty rates observed in Sri Lanka and Karnataka compared to Kerala. The Malabar Pit Viper is considered more dangerous than the HNPV, and HNPV is more potent in northern Kerala compared to the southern region. Juvenile snakes are more venomous than adults due to evolutionary pressures for survival." |  |
|  | | | | |  |
| Debates and Controversies (DC) | Controversies Over Tourniquet Use | DC-TQ | RPC | "Additionally, the use of a tourniquet can provide false reassurance to patients and bystanders, leading to patients arriving at the hospital without proper immobilisation." | 14 |
|  |  |  | FMS | "Training is essential for doctors to properly apply a tourniquet. When prompt medical care is available, using a tourniquet can often do more harm than good." |  |
|  |  |  | FMS | "Given that most areas are now well-connected by road, which minimises delays in reaching medical care, the use of tourniquets is generally not recommended. However, in remote areas where there may be delays in receiving medical attention, using a tourniquet might be considered if it helps prioritise life over limb." |  |
|  |  |  | FMS | "If a tourniquet is applied by an expert who has correctly identified a venomous bite, it can be beneficial in saving a life in remote settings without early accessibility to emergency care. Nevertheless, if there is swelling below the level of the tourniquet after its application, it raises the issue of whether antivenom should be administered, as it could be a non-venomous bite with swelling due to the tourniquet itself." |  |
|  |  |  | UB | "There are controversies regarding the application of tourniquets, but they can retard systemic absorption of venom. If used, the tourniquet should be applied to a single bone site, such as the thigh or arm, and should be neither too tight nor too loose. " |  |
|  |  |  | UB | "Do not remove the tourniquet until a decision about administering antivenom (ASV) has been made. It should be removed only when antivenom is started and flowing freely. " |  |
|  |  |  | KBM | "Applying a pressure bandage with optimal pressure, positioning, and width using a scientific approach can offer some benefits." |  |
|  |  |  | PKK | "For non-venomous snakebites, gangrene should not develop, so immobilisation of the affected area should be prioritised. The decision to use a tourniquet is complex and should be considered if medical help is delayed and there is expertise in its application. If a tourniquet is used, a blood pressure cuff can be helpful, and it is recommended to set up an IV line before removing the cuff." |  |
|  |  |  | FMS | "The issue arises when a patient arrives at the emergency department with a bite from an unknown snake species, which turns out to be non-venomous, and presents with local swelling above the tourniquet. The question then is whether antivenom (ASV) should be administered." |  |
|  |  |  | FMS | "Within the hospital, a blood pressure cuff can be applied proximal to the tourniquet and inflated above the patient's systolic blood pressure. Thereafter, the tourniquet can be removed, and the pressure cuff released slowly while the patient's vitals and blood picture are monitored." |  |
|  |  |  | UB | "It is very unusual for local swelling to occur with a non-venomous snakebite if the tourniquet is not applied too tightly. Tourniquets should not be used on distal sites." |  |
|  |  |  | SD | "The public often misunderstands instructions to apply a tourniquet, believing it should be tightened as much as possible, rather than maintaining the optimal pressure with a one-finger gap. This can result in severe complications, such as the need for amputation." |  |
|  |  |  | MA | "National guidelines advise against the use of tourniquets to ensure that a common protocol is followed, minimising the risk of doing more harm than good." |  |
|  |  |  | SVA | "In summary, if a tourniquet is to be used, avoid applying it as an arterial tourniquet. And if applied, it should only be applied by an expert." |  |
|  | Antivenom Use in Hump-Nosed Pit Viper | DC-HNPV | JK | "Recently, there has been an increase in cases involving Hump-Nosed Pit Vipers (HNPV) and Malabar Pit Vipers, in contrast to the more common Russell's Viper cases. Notably, antivenom serum (ASV) is ineffective against these species." | 5 |
|  |  |  | KBM | "The serious nature of pit viper bites was fully acknowledged in 2006, leading to a major shift in snakebite management practices." |  |
|  |  |  | KBM | "Recognising that ASV is ineffective for pit viper bites represents a major shift. This understanding has been crucial for minimising antivenom wastage, reducing the need for antivenom, and highlighting the importance of accurate snake species identification." |  |
|  |  |  | SD | "Diverse factors including ancestral origins, microhabitat preferences and prey-predator interactions of HNPV would have influenced its potency and the higher casualty rates observed in Sri Lanka and Karnataka compared to Kerala. The Malabar Pit Viper is considered more dangerous than the HNPV, and HNPV is more potent in northern Kerala compared to the southern region. Juvenile snakes are more venomous than adults due to evolutionary pressures for survival." |  |
|  |  |  | FMS | "In Southern Maharashtra, Karnataka, and Goa, Hump-Nosed Pit Vipers (HNPV) are causing more severe complications and higher mortality rates, with plasmapheresis often proving ineffective for these patients. Additionally, venom-induced consumption coagulopathy can lead to systemic manifestations such as stroke, myocardial infarction, and acute kidney injury." |  |
|  | Controversies in Plasmapheresis | DC-PLAS | JK | "Although the role of plasmapheresis in capillary leak syndrome is controversial in studies, we consider it when a patient develops bilateral parotid swelling, periorbital oedema, haemoconcentration, and refractory shock." | 5 |
|  |  |  | JK | "Capillary leak can occur in any organ, including the lungs, where leakage into the alveoli can result in high mortality, even when the patient is placed on a ventilator. The removal of toxins like Phospholipase A2, Zinc Metalloprotease, and Vascular Apoptosis-Inducing Proteins 1 & 2 (VAIP 1&2) via plasmapheresis shows promise in managing capillary leak syndrome." |  |
|  |  |  | AMR | "In some centres, plasmapheresis has been used when antivenom was ineffective or not indicated, and it has shown potential mortality benefits.” |  |
|  |  |  | IM | "Until recently, diagnosing capillary leak syndrome and determining the relevant parameters were unclear. Predicting and preventing this condition still requires further research and investigation. Currently, there are no methodological studies supporting the use of plasmapheresis for snakebite cases, so it is not yet recommended." |  |
|  |  |  | IM | "Comprehensive studies at the molecular level are needed to analyse the factors involved and the effects of plasmapheresis before it can be considered a viable treatment." |  |
|  | Blood Transfusion in Snakebite | DC-BT | JT | "Steroids are still administered for capillary leak syndrome, often based on cautious optimism." | 2 |
|  |  |  | JT | Blood component therapy is considered the next best option after administering the full dose of antivenom in cases of coagulopathy but using it without a clear indication can lead to more complications than benefits. |  |
|  | Issues in Snake Identification | DC-SI | KBM | "Initially thought to be a subspecies of the saw-scaled viper that did not respond to antivenom serum (ASV), the snake was later identified as a Hump-Nosed Pit Viper (HNPV). At that time, there was limited literature available to distinguish between these species." | 11 |
|  |  |  | KBM | "Recognising that ASV is ineffective for pit viper bites represents a major shift. This understanding has been crucial for minimising antivenom wastage, reducing the need for antivenom, and highlighting the importance of accurate snake species identification." |  |
|  |  |  | SD | "Diverse factors including ancestral origins, microhabitat preferences and prey-predator interactions of HNPV would have influenced its potency and the higher casualty rates observed in Sri Lanka and Karnataka compared to Kerala. The Malabar Pit Viper is considered more dangerous than the HNPV, and HNPV is more potent in northern Kerala compared to the southern region. Juvenile snakes are more venomous than adults due to evolutionary pressures for survival." |  |
|  |  |  | SD | "Options such as WhatsApp and Facebook groups, along with apps like Snakepedia and Sarpa, assist in snake identification. However, even for experts, identifying a snake without knowing its location can be challenging." |  |
|  |  |  | SD | "We maintain a WhatsApp group that tracks major snakebite cases in Kerala from the past 5-6 years. This data includes various snakebite cases, even from outside Kerala, and it remains to be analysed and published." |  |
|  |  |  | FMS | "The identification of snakebite species has become increasingly important, marking a significant shift in management practices." |  |
|  |  |  | IM | "The concern is whether the snake that bit the patient is the same as the one brought to the emergency room." |  |
|  |  |  | JK | "We can only confirm the species if the snake brought to the emergency is verified as the same one that bit the patient, and it was never out of sight after the bite." |  |
|  |  |  | FMS | "The biggest challenge arises when a bite isn't neutralised by ASV, and the initial decision is not to administer it. However, if the patient is transferred to another department, that decision might change." |  |
|  |  |  | PKK | "Effective communication is essential to explain that ASV is ineffective for snake bites from species other than the Big Four. Convincing patients that ASV won’t work and could cause additional complications is crucial." |  |
|  |  |  | RPC | "Identification of the snake, particularly juvenile forms, remains difficult even for experienced clinicians. Accurate initial identification is crucial as it influences the management approach." |  |
|  | Communication | DC-COM | PKK | "Effective communication is essential to explain that ASV is ineffective for snake bites from species other than the Big Four. Convincing patients that ASV won’t work and could cause additional complications is crucial." | 7 |
|  |  |  | IM | "The concern is whether the snake that bit the patient is the same as the one brought to the emergency room." |  |
|  |  |  | FMS | "The biggest challenge arises when a bite isn't neutralised by ASV, and the initial decision is not to administer it. However, when the patient is transferred to another department, that decision might change." |  |
|  |  |  | SD | "Sometimes, even the bystanders understand that ASV won't be effective, and they find themselves needing to convince the doctor not to administer it." |  |
|  |  |  | UB | "Administering ASV unnecessarily can lead to more complications, such as anaphylaxis, than any potential benefit to the patient." |  |
|  |  |  | FMS | "If a tourniquet is applied by an expert who has correctly identified a venomous bite, it can be beneficial in saving a life in remote settings without early accessibility to emergency care. Nevertheless, if there is swelling below the level of tourniquet after its application it raises the issue of whether antivenom should be administered, as it could be a non-venomous bite with swelling due to the tourniquet itself." |  |
|  |  |  | UB | There are controversies regarding application of tourniquet, but it can retard systemic absorption of venom. It is very useful if there is considerable delay in reaching a hospital where definitive treatment is available. When used, a tourniquet should be applied over a single bone site. Do not remove the tourniquet until a decision about administering antivenom (ASV) has been made. It should be removed only when antivenom is started and flowing freely. |  |
|  | | | | |  |
| Policy and Research (PR) | Policy Changes | PR-PC | MA | "National guidelines advise against the use of tourniquets to ensure that a common protocol is followed, minimising the risk of doing more harm than good." | 7 |
|  |  |  | MA | "The WHO aims to reduce snakebite deaths by 50% by 2030, which presents a significant challenge. The plan includes expanding the number of venom centres, with five regional centres already established and additional funding allocated." |  |
|  |  |  | MA | "There are concerns about the quality and efficacy of antivenom, as some are even distributed to African countries." |  |
|  |  |  | MA | "The strategy involves setting clear directions, timelines, and process indicators, followed by effective monitoring to improve snakebite identification and management." |  |
|  |  |  | RPC | "One major issue is the lack of notification, which means patients often present without any history of a snakebite, making diagnosis difficult, especially when symptoms are non-specific." |  |
|  |  |  | SD | "The public often misunderstands instructions to apply a tourniquet, believing it should be tightened as much as possible, rather than maintaining the optimal pressure with a one-finger gap. This can result in severe complications, such as the need for amputation." |  |
|  |  |  | UB | "It is very unusual for local swelling to occur with a non-venomous snakebite if the tourniquet is not applied too tightly. Tourniquets should especially not be used on distal sites. Training laypeople in tourniquet application can help minimise associated complications to an extent." |  |
|  | Future Research | PR-FR | UB | "Many pioneering faculty members collected substantial amounts of data, but much of it remained unanalysed. During this period, snakebite management was often neglected and limited to certain centres." | 11 |
|  |  |  | UB | "Experienced physicians observed that neostigmine had limited benefits for neurotoxic bites. In a well-equipped centre, neuroparalysis due to snakebite could be managed effectively by timely antivenom and artificial ventilation." |  |
|  |  |  | UB | "Antivenom production involves collecting venom, which can vary due to factors such as geographical location, seasonal changes, hibernation, and captivity. To address this, it is necessary to conduct multicentre production and comparison studies of antivenom to determine if region-specific antivenom should be developed." |  |
|  |  |  | JK | "There has been debate about how different brands of antivenom affect outcomes, particularly in relation to complications like capillary leak syndrome. It is essential to examine molecular-level inflammatory markers that contribute to capillary leak." |  |
|  |  |  | SD | "We maintain a WhatsApp group that tracks major snakebite cases in Kerala from the past 5-6 years. This data includes various snakebite cases, even from outside Kerala, and it remains to be analysed and published." |  |
|  |  |  | IM | "Until recently, diagnosing capillary leak syndrome and determining the relevant parameters were unclear.” |  |
|  |  |  | IM | “Predicting and preventing this condition still requires further research and investigation. Currently, there are no methodological studies supporting the use of plasmapheresis for snakebite cases, so it is not yet recommended." |  |
|  |  |  | IM | "Comprehensive studies at the molecular level are needed to analyse the factors involved and the effects of plasmapheresis before it can be considered a viable treatment." |  |
|  |  |  | IM | "Some inflammatory markers, such as matrix metalloproteinases (MMPs), are too small to be removed by plasmapheresis. However, we know that certain markers can activate the complement system, contributing to capillary leak syndrome." |  |
|  |  |  | KBM | "Ideally, antivenom should be produced for each individual snake species, and region-specific antivenom should be developed to ensure effective treatment. Regional specificity is important because antivenom may not neutralise every molecule, potentially leading to complications even if it targets the specific snake. Additionally, significant variation in venom toxicity—both in quality and quantity—occurs, with seasonal variations being particularly notable." |  |
|  |  |  | SD | "The public often misunderstands instructions to apply a tourniquet, believing it should be tightened as much as possible, rather than maintaining the optimal pressure with a one-finger gap. This can result in severe complications, such as the need for amputation." |  |

*All panellists were approached for authorship, and some non-panel contributors were included based on substantial contributions
