# Supplementary material for: Clinical challenges, controversies, and regional strategies in snakebite care in India
Source: Lancet Reg Health Southeast Asia. 2025 May 15;37:100598. doi: 10.1016/j.lansea.2025.100598 (PMC12145746; doi:10.1016/j.lansea.2025.100598)
Supplement: Supplementary Information S3 [file mmc3.docx]

## **Supplementary Information 3: Course outline**

**SAVIOR: Snakebite Assessment, Venomous bite Intervention, and Optimal Referral workshop**

Day One Agenda

| Sl.No | Time | Topic | Duration (mins) |
| --- | --- | --- | --- |
| 01 | 08.00-08.30 | Registration & Breakfast  Precourse Assessment | 30 |
| 02 | 08.30-08.45 | Introduction | 20 |
| 03 | 08.45-08.55 | One Health | 10 |
| 04 | 08.55-09.15 | Regional Epidemiology | 20 |
| 05 | 09.15-09.35 | Venom Composition and Pathophysiology of Envenomation | 20 |
| 06 | 09.35-09.55 | Conventional and Next Generation Snakebite Therapeutics | 20 |
| 07 | 10.00-10.30 | Inauguration Ceremony | 30 |
| 08 | 10.45-11.00 | Refreshments | 15 |
| 09 | 11.00 -11.15 | Prehospital Care | 15 |
| 10 | 11.15 -11.40 | Emergency Department Approach | 25 |
| 11 | 11.40 -12.00 | Lab Testing | 20 |
| 12a | 12.00-12.30 | Skill Station - 20 min Whole Blood Clotting Test/Modified Lee and White clotting test | 30 |
| 12b | 12.30-13.00. | Skill Station – Anti Snake Venom dilution | 30 |
| 13 | 13.00-13.20 | Ventilatory management | 20 |
| 14 | 13.20-14.00 | Lunch Break | 40 |
| 15a | 14.00-14.30 | Skill Station - Airway Management | 30 |
| 15b | 14.30-15.00 | Skill Station - Mechanical Ventilation | 30 |
| 16 | 15.00-15.25 | Capillary leak syndrome (CLS) | 25 |
| 17 | 15.25-15.50 | Surgical Management of Snakebite wounds | 25 |
| 18 | 15.50-16.00 | Tea Break | 10 |
| 19a | 16.00-16.30 | Skill Station- Fasciotomy | 30 |
| 19b | 16.30-17.00 | Skill Station- CLS | 30 |
| 20 | 17.00-17.25 | Optimal Referral | 25 |
| 21 | 17.25-17.45 | Debriefing & Closure | 20 |

Day Two Agenda

| Sl.No | Time | Topic | Duration (mins) |
| --- | --- | --- | --- |
| 01 | 08.00-08.30 | Breakfast | 30 |
| 02 | 08.30-09.00 | All Bites Approach | 30 |
| 03a | 09.00-09.45 | Newer Modalities - ROTEM | 45 |
| 03b | 09.45-10.30 | Newer Modalities - HBOT | 45 |
| 04 | 10.30-11.00 | Snakebite Simulation Scenario Practice | 30 |
| 05 | 11.00-11.20 | Refreshments | 20 |
| 06 | 11:20-13:00 | Snakebite Simulation Scenarios | 100 |
| 07 | 13.00-13.15 | Post Course Assessment | 15 |
| 08 | 13.15-14.00 | Lunch Break | 45 |
| 09 | 14.00-15.30 | Panel Discussion - Core Principles and Controversies in  Snakebite Management | 90 |
| 10 | 15.30-16.00 | Debriefing & Closure  Distribution of Certificates | 30 |

**SAFETi: Skills and Awareness for First aid, Emergency response, and wilderness Training**

| Sl.No | Time | Topic | Duration (mins) |
| --- | --- | --- | --- |
| 01 | 07.00-08.00 | Breakfast | 60 |
| 02 | 08.00-08.15 | Transport to Seminar Hall | 15 |
| 03 | 08.15-08.45 | Wilderness Assessment Triangle | 30 |
| 04 | 08.45-10.15 | Skills Demonstration & Practice | 30 |
| 05 | 10:15-10:30 | Refreshments | 15 |
| 06 | 10.30-12.00 | Into the Wild - Simulation Scenario | 90 |
| 07 | 12.00-12.30 | Scenario Debriefing | 30 |
| 08 | 12.30-01.30 | Lunch Break & Room Check Out | 45 |
| 09 | 01.30-02.30 | Toxic Plants | 30 |
| 10 | 03.00-03.20 | Bites & Stings | 20 |
| 11 | 03.20-03.40 | Water Disinfection | 20 |
| 12 | 03.40-04.00 | Debriefing & Closure | 30 |
