# Supplementary material for: Clinical challenges, controversies, and regional strategies in snakebite care in India
Source: Lancet Reg Health Southeast Asia. 2025 May 15;37:100598. doi: 10.1016/j.lansea.2025.100598 (PMC12145746; doi:10.1016/j.lansea.2025.100598)
Supplement: Supplementary Information S4 [file mmc4.docx]

### **Supplementary Information 4**

### **Detailed Coding Table and Thematic Analysis Framework**

**Title:** Coding and Thematic Analysis Process for the Study on Snakebite Management Challenges

### **1. Introduction**

This supplementary file provides a detailed account of the thematic analysis process employed in the study. It includes step-by-step descriptions of how codes were derived from the panel discussion data and linked to themes, demonstrating the transparency and rigour applied in the qualitative analysis. This document also outlines the framework and tools used to ensure methodological robustness.

### **2. Thematic Analysis Process**

The thematic analysis followed six systematic steps to transform raw panel discussion data into meaningful themes. Each step ensured consistency, transparency, and alignment with the study objectives.

#### **Step 1: Data Familiarisation**

- The panel discussion transcript was reviewed multiple times to ensure familiarity with the data. Each participant's statements were highlighted and segmented into meaningful units relevant to the research objectives. Verbatim quotes were noted for initial coding.

**Example:**

Quote: "Recognising that ASV is ineffective for pit viper bites represents a major shift."
**Notes:** Highlighted as a significant paradigm shift in antivenom use.

#### **Step 2: Initial Coding**

- Short descriptive labels (codes) were assigned to segments of data that captured key ideas or recurring concepts.
- Codes were derived inductively based on the content of the transcript and deductively from predefined research objectives.
- The transcript was initially coded by AK. The codes were verified by AS and DM.
- Subsequent discussion and reassignment of codes were done by SVA and DM.

**Example:**

| **Verbatim Quote** | **Initial Code** | **Justification** |
| --- | --- | --- |
| "Capillary leak can occur in any organ, including the lungs, where leakage into the alveoli can result in high mortality." | CP-CLS | Captures clinical challenges associated with capillary leak syndrome. |
| "The serious nature of pit viper bites was fully acknowledged in 2006, leading to a major shift." | ES-AV | Reflects historical changes in antivenom use and dosage practices. |
| "National guidelines advise against the use of tourniquets to ensure that a common protocol is followed." | DC-TQ | Highlights debates over the use of tourniquets in snakebite management. |

#### **Step 3: Aggregation and Refinement of Codes**

Codes were reviewed, refined, and grouped into subthemes to ensure clarity and consistency. The codes were reviewed and grouped into categories based on their conceptual similarity. Redundant or overlapping codes were consolidated, and ambiguous codes were clarified through core team discussions (SVA, DM, AS, AG, VC). The decision of SVA was taken as final.

**Examples:**

| **Initial Code** | **Refined Code** | **Rationale** |
| --- | --- | --- |
| "Historical changes in ASV dosage" | ES-AV | Consolidated under the broader category of historical changes in antivenom use. |
| "Capillary leak complications" | CP-CLS | Unified under the clinical challenge of capillary leak syndrome. |

#### **Step 4: Theme Development**

Themes were developed by grouping related codes and identifying patterns across the data. These themes were refined through iterative discussions among the researchers.

Subthemes were grouped into broader themes based on conceptual similarity and relevance to the research objectives. The themes were discussed with all co authors. And suggestions and changes incorporated.

Themes were refined through iterative discussions among the core research team.

Theme maps were made manually first. Each theme was circled each time it was repeated and connected with themes it merged or interacted with.

Overarching themes were derived through inductive reasoning

**Example Table of Theme Development:**

| **Codes** | **Subtheme** | **Theme** | **Justification** |
| --- | --- | --- | --- |
| ES-AV, DC-HNPV, DC-SI | Historical changes in antivenom use | Evolution of Snakebite Treatment Paradigms | Captures shifts in practices, debates over antivenom use, and the importance of accurate snake species identification. |
| CP-CLS, CP-C, CP-ST | Clinical challenges in snakebite management | Clinical and Procedural Challenges | Highlights operational and clinical difficulties, such as managing capillary leak syndrome and delays in patient presentation. |
| DC-TQ, DC-PLAS, DC-BT | Debates over treatment protocols | Debates and Controversies | Discusses contested treatment protocols, including tourniquet use, plasmapheresis, and blood transfusion. |

#### **Step 5: Frequency summaries**

Frequency summaries were generated to provide an overview of recurring themes while maintaining a focus on thematic depth and relevance. They were generated to enhance transparency and provide context regarding the relative emphasis on themes during the discussion and not used to prioritise or rank themes. They served as an adjunct to the interpretative analysis, which focused on relational dynamics, cross-cutting themes, and actionable insights.

| **Theme** | **Number of Codes** | **Frequency of Related Quotes** | **Depth/Impact Justification** |
| --- | --- | --- | --- |
| Evolution of Snakebite Treatment Paradigms | 3 | 12 | Captures significant shifts in snakebite management practices over decades. |
| Clinical and Procedural Challenges | 5 | 18 | Includes critical issues such as capillary leak syndrome and inconsistencies in care delivery. |
| Debates and Controversies | 6 | 14 | Highlights unresolved controversies and areas requiring further research and policy action. |

**Frequency Summary**

While the thematic analysis process included documenting the frequency of topics discussed during the panel, these counts were not used to rank or prioritise themes. Instead, they provided a contextual overview of the relative emphasis placed on various aspects of snakebite management. This approach allowed us to identify key focus areas of the panel discussion while ensuring that the interpretative analysis remained central to our findings. The themes were derived through an iterative process of coding, aggregation, and interpretative synthesis, with the frequency data complementing the qualitative insights rather than defining them. This framework emphasised the relational dynamics between themes and the depth of insights shared during the discussion.

Theme 1: Evolution of Snakebite Treatment Paradigms (ES); n=3 (KBM, UB, PKK) focused on the historical evolution of antivenom use (n=4), advancements in specialised care (n=2), and the challenges in early diagnosis (n=3), giving a total frequency of 9. Theme 2: Clinical and Procedural Challenges (CP); n=3 highlighted various aspects, including challenges in snakebite management (n=15), paediatric snakebite management (n=2), allergy and anaphylaxis (n=3), venom-induced coagulopathy (n=11), thrombotic microangiopathy (n=4), stroke in snakebite cases (n=9), capillary leak syndrome (n=12), and regional differences in venom and antivenom potency (n=10), with a total frequency of 66. Theme 3: Debates and Controversies (DC) covered key areas of contention, including tourniquet use (n=14), antivenom for *H*. *hypnale* (n=5), controversies in plasmapheresis (n=5), blood transfusion in snakebite (n=2), issues in snake identification (n=11), and communication (n=7), resulting in a total frequency of 44. Theme 4: Policy and Research (PR); n=2 discussed the importance of policy changes (n=7) and the need for future research (n=11), with a total frequency of 18.

**Step 6: Thematic map generation**

A thematic map was created using draw.io to visualise the relationships between themes and subthemes by SVA. The core committee suggested usage of a thematic map to better understand theme interactions and generate overarching themes. The thematic map was developed using the draw.io platform to visually represent the relationships between themes and subthemes identified during the analysis. Major themes were depicted with thick-bordered boxes, while subthemes were shown with thin borders, with the border thickness of each subtheme proportional to the frequency of discussion. Interactions between themes and subthemes were denoted using arrows: solid arrows indicated connections between major themes, dashed arrows connected subthemes, and curved arrows represented intertheme interactions. Each theme was colour-coded for clarity: purple for Evolution of Snakebite Treatment Paradigms (ES), red for Challenges in Practice (CP), orange for Debates and Controversies (DC), blue for Policy and Research (PR), and dark red for the SAVIOR Panel Discussion, which served as the central connector. To ensure comprehensive representation, sentences relevant to multiple themes were duplicated and assigned to all applicable codes. The strength of connections was visually represented by arrow thickness, with thicker arrows indicating stronger interactions and thinner arrows indicating weaker links. This structured approach allowed for an intuitive and systematic depiction of thematic relationships derived from the data.

Overarching themes were identified after in-depth discussions, repeated exposure to the data to identify patterns, nuances, and relationships between codes.

####

#### **Step 6: Linking Codes to Verbatim Quotes**

To ensure transparency, selected quotes were explicitly linked to their corresponding codes and themes.

**Example Table of Code-Quote-Theme Mapping:**

| **Verbatim Quote** | **Code** | **Theme** | **Remarks** |
| --- | --- | --- | --- |
| "Recognising that ASV is ineffective for pit viper bites represents a major shift." | ES-AV | Evolution of Snakebite Treatment Paradigms | Highlights a paradigm shift in the use of antivenom for non-Big Four species, reducing waste…. may be discus about advances in specialised care? |
| "Capillary leak can occur in any organ, including the lungs, where leakage into the alveoli can result in high mortality." | CP-CLS | Clinical and Procedural Challenges | Demonstrates procedural challenges and mortality drivers in hemotoxic bites. |
| "National guidelines advise against the use of tourniquets to ensure that a common protocol is followed." | DC-TQ | Debates and Controversies | Illustrates conflicting perspectives on tourniquet use and its implications for national guidelines. |

#### **Step 6: Validation and Reflexivity**

- **Inter-Coder Reliability:** Coding was independently performed by two researchers and cross-verified by a third.
- **Reflexive Practices:** Reflexive notes were maintained to mitigate biases, and themes were reviewed iteratively.
- **Participant Validation:** Panellists were provided with themes and supporting quotes to ensure accuracy and authenticity.

Data collection involved video recordings of the 90-minute panel discussion using a Canon EOS 60D camera, with a Canon EOS 1500D as backup. Audio and video were stored as MP4 files and securely backed up. Field notes were taken during the session using Microsoft OneNote on a Surface Pro. Audio recordings were transcribed verbatim by AG, verified by DM, and cross-checked for accuracy by VC. The transcripts were shared with panellists for revision to ensure accuracy. Initial coding of the transcripts was done using Microsoft Excel, with additional codes generated using OpenAI's GPT-4 and manually assigned by AG and SVA, then verified by AS.

### **4. Codebook**

A structured codebook was developed to ensure consistency in the coding process. Below is an example entry:

| **Code** | **Definition** | **Example Quote** |
| --- | --- | --- |
| **Ineffectiveness of ASV** | Refers to instances where antivenom serum is reported to be ineffective against specific snake species. | "Recognising that ASV is ineffective for pit viper bites represents a major shift in management." |
| **Capillary leak syndrome (CLS)** | Refers to the condition caused by hemotoxic venom, leading to increased vascular permeability. | "Early recognition of capillary leak syndrome remains challenging." |
| **Tourniquet use controversy** | Refers to differing opinions on the utility and safety of tourniquets in snakebite management. | "Given that most areas are now well-connected, the use of tourniquets is generally not recommended." |

##

**Coded Transcript**

**Dr Siju > Dr K B Mohan:**

**Question:** Could you please share the insights on how the treatment paradigms for snakebite have changed throughout your career?

**Dr. K B Mohan:**

- "In the mid-1970s, antivenom was limited and typically administered in low doses, with a standard treatment of around 10 vials regardless of the snake species. At that time, it was believed that this dosage was adequate to counteract the venom of the 'big four' species." **(ES- AV, CP-C)**
- "The serious nature of pit viper bites was fully acknowledged in 2006, leading to a major shift in snakebite management practices." **(ES- AV, DC-HNPV)**
- "Recognising that ASV is ineffective for pit viper bites represents a major shift. This understanding has been crucial for minimising antivenom wastage, reducing the need for antivenom, and highlighting the importance of accurate snake species identification." **(DC- HNPV, ES- AV, DC-SI)**
- "In 1979, a specialised critical care unit for snakebite cases was established at Calicut Medical College in response to high mortality rates from complications like capillary leak syndrome, acute renal injury, and cardiorespiratory and neurological issues. The introduction of hemodialysis and ventilatory support in these units greatly reduced mortality rates." **(ES-SC,CP-CLS )**

**Dr Siju > Dr Udayabhaskaran:**

**Question:** Could you share some challenges from the early days in the management of snakebite and establishment of protocols?

**Dr. Udayabhaskaran:**

- "The limited number of clinical experts specialising in snakebite management created a significant burden on those in the field. Additionally, there was a lack of local dialysis facilities for treating snakebite cases so that a person was sent to Chennai for training in dialysis**.**" **(ES-SC, ES-DX)**
- "Many pioneering faculty members collected substantial amounts of data, but much of it remained unanalysed. During this period, snakebite management was often neglected and limited to certain centres." **(ES- DX, PR-FR)**
- "Experienced physicians observed that neostigmine had limited benefits for neurotoxic bites. In a well-equipped centre, neuroparalysis due to snakebite could be managed effectively by timely antivenom and artificial ventilation. **(PR-FR)**

**Dr Siju > Dr. Jayesh:**

**Question:** What are the unique challenges of managing snake bites at a high-volume centre?

**Dr. Jayesh:**

- "There is considerable variation in the presentation of individual snakebite cases, often compounded by delays in reaching the hospital after a bite and inconsistencies in management at referral hospitals." **(CP-C)**
- "Recently, there has been an increase in cases involving Hump-Nosed Pit Vipers (HNPV) and Malabar Pit Vipers, in contrast to the more common Russell's Viper cases. Notably, antivenom serum (ASV) is ineffective against these species."**(CP-RD, DC- HNPV)**
- "An increase in the toxicity and complications associated with hemotoxic bites has been observed. Early recognition of capillary leak syndrome, the primary cause of mortality in hemotoxic bites, remains challenging. Despite the use of ASV and supportive care, mortality due to capillary leak is high, even after correcting coagulation parameters." **(CP-CLS)**
- "Capillary leak can occur in any organ, including the lungs, where leakage into the alveoli can result in high mortality, even when the patient is placed on a ventilator. The removal of toxins like Phospholipase A2, Zinc Metalloprotease, and Vascular Apoptosis-Inducing Proteins 1 & 2 (VAIP 1&2) via plasmapheresis shows promise in managing capillary leak syndrome." **(CP-CLS, DC-PLAS)**
- "Cases have demonstrated a seasonal variation in the occurrence of capillary leak, further complicating management strategies." **(CP-CLS)**
- "The non-specific nature of snakebite symptoms presents a significant challenge; for example, Cobra bites have been linked to cardiomyopathy, sparking debate over the potential use of beta blockers in treatment." (Controversies in Treatment Approaches) **(CP-C)**

**Dr. Purushothaman > Dr. Jayesh:**

**Question:** Although not typically recommended, should we consider using plasmapheresis for treating capillary leak syndrome?

**Dr. Jayesh:**

- "Although the role of plasmapheresis in capillary leak syndrome is controversial in studies, we consider it when a patient develops bilateral parotid swelling, periorbital oedema, haemoconcentration, and refractory shock." **(CP-CLS, DC- PLAS)**

**Dr Siju > Dr Rafi:**

**Question:** Could you elaborate on thrombotic microangiopathy in snake bite cases?

**Dr. Rafi:**

- "Thrombotic microangiopathy (TMA) is not uncommon in snakebite cases in India, often presenting with atypical symptoms. Diagnosis is confirmed by identifying schistocytes, thrombocytopenia, and microangiopathic haemolytic anaemia, with acute kidney injury (AKI) being the most common form of end-organ damage." **(CP-TMA)**
- "In some centres, plasmapheresis has been used when antivenom was ineffective or not indicated, and has shown potential mortality benefits." **(CP-TMA, DC- PLAS)**

**Dr Siju > Dr. Freston:**

**Question:** Could you provide your thoughts on venom-induced consumption coagulopathy?

**Dr. Freston:**

- "The identification of snakebite species has become increasingly important, marking a significant shift in management practices." **(DC-SI, ES-AV)**
- "In Southern Maharashtra, Karnataka, and Goa, Hump-Nosed Pit Vipers (HNPV) are causing more severe complications and higher mortality rates, with plasmapheresis often proving ineffective for these patients. Venom-induced consumption coagulopathy can lead to systemic manifestations such as stroke, myocardial infarction, and acute kidney injury." **(CP-C, CP-VIC, CP-ST, DC-HNPV, CP-RD, CP-TMA)**

**Dr Siju > Dr. Purushothaman:**

**Question:** What are the diagnostic challenges for paediatric snake bite cases and how do we approach them differently?

**Dr. Purushothaman:**

- "The response of paediatric patients to treatments or interventions can vary somewhat, and even experienced faculty have limited experience with cases involving newborns." **(CP-PED)**
- "More snakebites occur at night, these are often less frequently diagnosed. The challenge is exacerbated by the painless bites of the common krait." **(CP-C)**
- "Many snakebites happen in school settings, and it is recommended to use smaller volumes of treatment, generally 5-10 ml/kg." **(CP-PED)**

**Dr. Purushothaman > Dr. Sandeep:**

**Question:** Is it possible for the venom toxins in India to evolve and exhibit more thrombotic features similar to those seen in Hump-Nosed Pit Vipers in Sri Lanka?

**Dr. Sandeep:**

- "Diverse factors including ancestral origins, microhabitat preferences and prey-predator interactions of HNPV would have influenced its potency and the higher casualty rates observed in Sri Lanka and Karnataka compared to Kerala. The Malabar Pit Viper is considered more dangerous than the HNPV, and HNPV is more potent in northern Kerala compared to the southern region. Juvenile snakes are more venomous than adults due to evolutionary pressures for survival." **(DC- HNPV, DC- SI, CP-RD)**
- "We maintain a WhatsApp group that tracks major snakebite cases in Kerala from the past 5-6 years. This data includes various snakebite cases, even from outside Kerala, and it remains to be analysed and published." **(DC- SI, PR-FR)**

**Dr Jayesh:**

- "There has been debate about how different brands of antivenom affect outcomes, particularly in relation to complications like capillary leak syndrome. It is essential to examine molecular-level inflammatory markers that contribute to capillary leak." **(PR-FR, CP-CLS)**

**Dr. Indira:**

- "Until recently, diagnosing capillary leak syndrome and determining the relevant parameters were unclear. Predicting and preventing this condition still requires further research and investigation. Currently, there are no methodological studies supporting the use of plasmapheresis for snakebite cases, so it is not yet recommended." **(CP-CLS, DC- PLAS, PR-FR)**
- "Comprehensive studies at the molecular level are needed to analyse the factors involved and the effects of plasmapheresis before it can be considered a viable treatment." **( CP-CLS, PR-FR, DC-PLAS)**
- "Some inflammatory markers, such as matrix metalloproteinases (MMPs), are too small to be removed by plasmapheresis. However, we know that certain markers can activate the complement system, contributing to capillary leak syndrome." **(CP-CLS, PR-FR)**

**Dr Udayabhaskaran:**

- "Antivenom production involves collecting venom, which can vary due to factors such as geographical location, seasonal changes, hibernation, and captivity. To address this, it is necessary to conduct multicentre production and comparison studies of antivenom to determine if region-specific antivenom should be developed." **(CP-RD, PR- FR)**

**Dr KB Mohan:**

- "Ideally, antivenom should be produced for each individual snake species, and region-specific antivenom should be developed to ensure effective treatment. Regional specificity is important because antivenom may not neutralise every molecule, potentially leading to complications even if it targets the specific snake. Additionally, significant variation in venom toxicity—both in quality and quantity—occurs, with seasonal variations being particularly notable." **(CP-RD, PR- FR)**

**Dr. Jayesh:**

- "Institutions are using various brands of antivenom, highlighting the need to compare these brands to assess their efficacy and associated complications." **(CP-RD)**

**Dr. Joe:**

- "Some centres face storage issues with liquid vials, which require a temperature range of 2-8°C. Inconsistent adherence to these storage requirements can impact efficacy." **(CP-RD)**
- "Steroids are still administered for capillary leak syndrome, often based on cautious optimism. Blood component therapy is considered the next best option after administering the full dose of antivenom in cases of coagulopathy, but using it without a clear indication can lead to more complications than benefits." **(CP-CLS, DC-BT, CP-VIC)**

**Dr. Siju > Dr. Manu:**

**Question:** What are your thoughts on policy changes related to region-specific antivenom production and management strategies? How will NAPSE contribute to these improvements?

**Dr. Manu:**

- "There are concerns about the quality and efficacy of antivenom, as some are even distributed to African countries." **(CP-RD, PR-PC)**
- "The WHO aims to reduce snakebite deaths by 50% by 2030, which presents a significant challenge. The plan includes expanding the number of venom centres, with five regional centres already established and additional funding allocated. There is also a push for states to develop state action plans and districts to create district action plans." **(PR-PC)**
- “The strategy involves setting clear directions, timelines, and process indicators, followed by effective monitoring to improve snakebite identification and management. Districts receive direct funds with oversight for training, while state-level initiatives are crucial. For example, Karnataka has made snakebite cases notifiable. These strategic plans are designed to provide clear guidance on what needs to be done over the next decade.” **(PR- PC, CP-RD)**

**Dr. Siju > Dr. Rajeev:**

**Question:** As an emergency physician, what are the main challenges you encounter, particularly with referred patients?

**Dr. Rajeev:**

- "One major issue is the lack of notification, which means patients often present without any history of a snakebite, making diagnosis difficult, especially when symptoms are non-specific." **(CP-C, PR-PC)**
- "Delays in seeking emergency care further complicate matters; patients may visit traditional healers, who might send them home without observing warning signs, not considering the possibility of delayed symptoms."**(CP-C)**
- "Another challenge is the lack of resuscitation support at peripheral centres." **(CP-C)**
- "Sometimes, antivenom (ASV) may not be administered even when needed due to concerns about anaphylaxis or confusion about its indications. There is also significant confusion and inconsistency regarding the appropriate dosage of ASV for different snakebites." **(CP-RD, CP-C, CP-AX)**
- "Identification of the snake, particularly juvenile forms, remains difficult even for experienced clinicians. Accurate initial identification is crucial as it influences the management approach." **(DC-SI)**
- "Additionally, the use of a tourniquet can provide false reassurance to patients and bystanders, leading to patients arriving at the hospital without proper immobilisation." **(DC-TQ, CP-C)**

**Dr. Purushothaman**

- "Of course, the main highlight is strengthening the peripheral unit for early stabilisation and administration of ASV. However, by the time they reach tertiary centres, complications have already set in." **( CP-C)**

**Audience**

- "All snakebites we refer to the medical college. That's safest for us and the patient. We are ill-equipped to take care of snakebites."

**Dr. Siju:** What is the panel's perspective on the use of tourniquets in snakebite cases? First let me ask what is your experience with Pressure Immobilisation technique? Have you seen it?

**Dr. Freston:**

- "Training is essential for doctors to properly apply a tourniquet. When prompt medical care is available, using a tourniquet can often do more harm than good." **(DC-TQ)**
- "Given that most areas are now well-connected by road, which minimises delays in reaching medical care, the use of tourniquets is generally not recommended. However, in remote areas where there may be delays in receiving medical attention, using a tourniquet might be considered if it helps prioritise life over limb." **(DC-TQ, CP-C)**
- "If a tourniquet is applied by an expert who has correctly identified a venomous bite, it can be beneficial in saving a life in remote settings without early accessibility to emergency care. Nevertheless, if there is swelling below the level of tourniquet after its application it raises the issue of whether antivenom should be administered, as it could be a non-venomous bite with swelling due to the tourniquet itself." **(DC-TQ, DC-COM)**

**Dr. Udayabhaskaran:**

- There are controversies regarding application of tourniquet, but it can retard systemic absorption of venom. It is very useful if there is considerable delay in reaching a hospital where definitive treatment is available. If used, the tourniquet should be applied to a single bone site, such as the thigh or arm, and should be neither too tight nor too loose. Do not remove the tourniquet until a decision about administering antivenom (ASV) has been made. It should be removed only when antivenom is started and flowing freely. **(DC-TQ, DC-COM)**

**Dr. KB Mohan:**

- "Applying a pressure bandage with optimal pressure, positioning, and width using a scientific approach can offer some benefits."**(DC-TQ)**

**Dr. Purushothaman:**

- "For non-venomous snakebites, it is inappropriate to develop gangrene, so immobilisation of the affected area should be prioritised. The decision to use a tourniquet is complex and should be considered if medical help is delayed and there is expertise in its application. If a tourniquet is used, a blood pressure cuff can be helpful, and it is recommended to set up an IV line before removing the cuff." **(DC-TQ)**

**Dr. Freston:**

- "The issue arises when a patient arrives at the emergency department with a bite from an unknown snake species, which turns out to be non-venomous, and presents with local swelling distal to the the tourniquet. The question then is whether antivenom (ASV) should be administered." **(CP-C, DC-TQ)**

**Dr. Udayabhaskaran:**

- "It is very unusual for local swelling to occur with a non-venomous snakebite if the tourniquet is not applied too tightly. Tourniquets should especially not be used on distal sites. Training laypeople in tourniquet application can help minimise associated complications to an extent." **(DC-TQ, PR-PC)**

**Dr. Freston:**

- "Within the hospital, a blood pressure cuff can be applied proximal to the tourniquet applied and inflated above the patient's systolic blood pressure. Thereafter the tourniquet can be removed and the pressure cuff released slowly while the patient's vitals and blood picture can be monitored." **(DC-TQ)**

**Dr. Sandeep:**

- "The public often misunderstands instructions to apply a tourniquet, believing it should be tightened as much as possible, rather than maintaining the optimal pressure with a one-finger gap. This can result in severe complications, such as the need for amputation." **(DC-TQ, PR-FR,PR-PC)**

**Dr. Manu:**

- "National guidelines advise against the use of tourniquets to ensure that a common protocol is followed, minimising the risk of doing more harm than good. " **(DC-TQ, PR-PC)**

**Dr. Siju:**

- "In summary, if a tourniquet is to be used, avoid applying it as an arterial tourniquet. And if applied, it should only be applied by an expert." **(DC-TQ)**

**Dr. Siju:** How do considerations about the risk of anaphylaxis from antivenom, along with the availability of ventilators and airway management, impact peripheral centres’ decisions to administer antivenom?

**Dr. Freston:**

- "Anaphylaxis can occur with any amount of antivenom (ASV). Therefore, the full required dose should be given and the patient monitored before transfer. "**(CP-AX, CP-C)**

**Dr. Siju:** What are the panelists' views on administering ASV for venomous bites from snakes other than the Big Four, such as HNPV?

**Dr. Purushothaman:**

- "Effective communication is essential to explain that ASV is ineffective for snake bites from species other than the Big Four. Convincing patients that ASV won’t work and could cause additional complications is crucial." **(DC-COM, DC-SI)**

**Dr. Indira:**

- "The concern is whether the snake that bit the patient is the same as the one brought to the emergency room." **(DC-SI, DC-COM)**

**Dr. Freston:**

- "The biggest challenge arises when a bite isn't neutralised by ASV, and the initial decision is not to administer it. However, if the patient is transferred to another department, that decision might change. **" (DC-COM, DC-SI)**

**Dr. Sandeep:**

- "Sometimes, even the bystanders understand that ASV won't be effective, and they find themselves needing to convince the doctor not to administer it." **(DC-COM)**

**Dr. Udayabhaskaran:**

- "Administering ASV unnecessarily can lead to more complications, such as anaphylaxis, than any potential benefit to the patient." **(CP-AX, DC-COM)**

**Dr. Jayesh:**

- "We can only confirm the species if the snake brought to the emergency is verified as the same one that bit the patient, and it was never out of sight after the bite." **(DC-SI)**

**Audience:** Is delayed stroke presentation common in cases of vasculotoxic snake bites?

**Dr. Udayabhaskaran:**

- "Since snake venom contains both procoagulants and anticoagulants, the dominant factors determine the presentation of symptoms." **(CP-ST, CP- VIC)**

**Dr. Purushothanam:**

- "We need to study the coagulant and anticoagulant properties of each snake species individually. For example, the Malabar pit viper exhibits a stronger procoagulant effect than anticoagulant, leading to a higher likelihood of stroke." **(CP-ST, CP- VIC)**

**Dr. Udayabhaskaran:**

- "In cases of hemispheric stroke, which is typically a major vessel embolic stroke, is anticoagulation an appropriate treatment option for snake bite cases?" **(CP-ST, CP- VIC)**

**Dr. Rafi:**

- "The use of anticoagulation for patients with embolic stroke caused by vasculotoxic snake bites remains controversial." **(CP-ST, CP-VIC)**

**Dr. Jayesh:**

- "Although initial thromboembolic phenomena may be observed, patients can later develop haemorrhagic issues. Early thrombolysis might worsen the patient's clinical condition." **(CP-ST, CP- VIC)**

**Dr. Rajeev:**

- "Strokes in snake bite cases are multifactorial and can result from thromboembolic events or watershed infarctions caused by hypotension." **(CP-ST, CP- VIC, CP- TMA, CP- CLS)**

**Dr. Siju:**

- "Over the past three years, there have been seven cases of ischemic infarcts associated with snake bites in Jubilee Mission Medical College, Thrissur." **(CP-ST, CP-VIC)**

**Dr. K B Mohan:**

- "Initially thought to be a subspecies of the saw-scaled viper that did not respond to antivenom serum (ASV), the snake was later identified as a Hump-Nosed Pit Viper (HNPV). At that time, there was limited literature available to distinguish between these species." **(ES-DX, DC-SI)**

**Dr. Sandeep:**

- "Options such as WhatsApp and Facebook groups, along with apps like Snakepedia and Sarpa, assist in snake identification. However, even for experts, identifying a snake without knowing its location can be challenging." **(DC-SI)**
