# Supplementary material for: Clinical challenges, controversies, and regional strategies in snakebite care in India
Source: Lancet Reg Health Southeast Asia. 2025 May 15;37:100598. doi: 10.1016/j.lansea.2025.100598 (PMC12145746; doi:10.1016/j.lansea.2025.100598)
Supplement: Supplementary Information S5 [file mmc5.docx]

### **Supplementary Information 5**

### **National Action Plan for Prevention and Control of Snakebite Envenoming (NAPSE)**

The **National Action Plan for Prevention and Control of Snakebite Envenoming (NAP-SE)**, developed by the **Centre for One Health** under the **National Centre for Disease Control (NCDC)**, aims to reduce snakebite-related deaths and disabilities in India by half by 2030. The full guidance document is available at [NAP-SE](https://ncdc.mohfw.gov.in/wp-content/uploads/2024/07/NATIONAL-ACTION-PLAN-FOR-PREVENTION-AND-CONTROL-OF-SNAKEBITE-ENVENOMING-NAPSE.pdf). NAP-SE serves as a strategic framework to guide state and district-level stakeholders in creating tailored action plans for the prevention, control, and management of snakebite envenoming.

#### **Objectives and Strategic Priorities**

Key objectives of NAP-SE include:

- Reducing morbidity, mortality, and associated complications due to snakebite envenoming.
- Enhancing healthcare capacity through training, public awareness, and optimised referral mechanisms.
- Ensuring sustained availability of anti-snake venom (ASV) and establishing regional venom centres.
- Promoting operational research on anti-venoms and snakebite diagnostics.

The plan’s scope extends beyond healthcare facilities, involving wildlife, veterinary, and tribal health sectors. It emphasises intersectoral collaboration, community outreach, and public-private partnerships to create a holistic approach to snakebite management.

#### **The SBLS Workshop as a Pilot for NAP-SE Implementation**

The **Snake Bite Life Support (SBLS)** workshop served as a practical pilot program for implementing NAP-SE recommendations. Aligned with NAP-SE priorities, the workshop focused on:

1. **Capacity Building**: Training healthcare workers in first aid, clinical diagnosis, and management of snakebites, including early antivenom administration and managing complications like anaphylaxis.
2. **Referral Optimisation**: Enhancing coordination between primary and tertiary healthcare systems for the timely transfer of critical patients.
3. **Community Engagement**: Raising public awareness about snakebite prevention, safety measures, and the importance of immediate medical attention.

#### **Key Areas Addressed in the SBLS Workshop**

Specific areas of the SBLS workshop directly address NAP-SE priorities:

- **First-Responder Training**: Equipping first responders with knowledge and skills to stabilise snakebite victims and prevent delays in care.
- **Clinical Management Strategies**: Standardising protocols for antivenom administration, managing coagulopathy, and addressing neurotoxic symptoms.
- **Public Health Outreach**: Educating communities on snakebite prevention, identification, and safe practices to minimise human-snake conflict.

#### **Conclusion**

The SBLS workshop demonstrated the feasibility of NAP-SE’s strategic framework, showcasing measurable improvements in healthcare workers’ competence and the efficiency of referral systems. By incorporating the lessons learned from the SBLS initiative, NAP-SE provides a robust roadmap for reducing snakebite-related morbidity and mortality nationwide. This expanded supplementary section highlights the synergistic relationship between the SBLS workshop and NAP-SE, ensuring that the latter’s objectives are translated into actionable outcomes.

Further details on NAP-SE, including the full action plan, are available at<https://ncdc.mohfw.gov.in/wp-content/uploads/2024/07/NATIONAL-ACTION-PLAN-FOR-PREVENTION-AND-CONTROL-OF-SNAKEBITE-ENVENOMING-NAPSE.pdf>.
